# Abscisic Acid Regulates the Root Growth Trajectory by Reducing Auxin Transporter *PIN2* Protein Levels in *Arabidopsis thaliana*

**DOI:** 10.3389/fpls.2021.632676

**Published:** 2021-03-04

**Authors:** Qijun Xie, Jemaa Essemine, Xiaochen Pang, Haiying Chen, Jing Jin, Weiming Cai

**Affiliations:** ^1^Laboratory of Photosynthesis and Environment, CAS Centre for Excellence in Molecular Plant Sciences, Shanghai Institute of Plant Physiology and Ecology, Chinese Academy of Sciences, Shanghai, China; ^2^State Key Laboratory of Chemo/Biosensing and Chemometrics, College of Biology, Hunan Key Laboratory of Plant Functional Genomics and Developmental Regulation, Hunan University, Changsha, China; ^3^National Key Laboratory of Plant Molecular Genetics, CAS Centre for Excellence in Molecular Plant Sciences, Shanghai Institute of Plant Physiology and Ecology, Chinese Academy of Sciences, Shanghai, China

**Keywords:** abscisic acid, PIN-FORMED, shootward auxin transport, vertical growth index, root growth trajectory, agravitropism-like

## Abstract

The root is in direct contact with soil. Modulation of root growth in response to alterations in soil conditions is pivotal for plant adaptation. Extensive research has been conducted concerning the adjustment of root elongation and architecture in response to environmental factors. However, little is known about the modulation of the root growth trajectory, as well as its hormonal mechanism. Here we report that abscisic acid (ABA) participated in controlling root growth trajectory. The roots upon ABA treatment or from ABA-accumulation double mutant *cyp707a1,3* exhibit agravitropism-like growth pattern (wavy growth trajectory). The agravitropism-like phenotype is mainly ascribed to the compromised shootward transportation of auxin since we detected a reduced fluorescence intensity of auxin reporter DR5:VENUS in the root epidermis upon exogenous ABA application or in the endogenous ABA-accumulation double mutant *cyp707a1,3*. We then tried to decipher the mechanism by which ABA suppressed shootward auxin transport. The membrane abundance of PIN2, a facilitator of shootward auxin transport, was significantly reduced following ABA treatment and in *cyp707a1,3*. Finally, we revealed that ABA reduced the membrane PIN2 intensity through suppressing the *PIN2* expression rather than accelerating PIN2 degradation. Ultimately, our results suggest a pivotal role for ABA in the root growth trajectory and the hormonal interactions orchestrating this process.

## Introduction

Due to their sessile properties, the organ growth of plants exhibits plasticity. Since the root is located in a direct contact with the soil environment, its growth plasticity is indispensable for the plant adaptation. At least three parameters describe root growth: growth/elongation rate, architecture and growth trajectory/direction (e.g., grow straight or flexuous). Root growth concerning the 3 parameters varies frequently in the face of unstable soil environments. For instance, when roots encounter soil drought, the growth rate of the primary root will be significantly accelerated to search for water in deep soil (Xu et al., 2013). When the soil has a low nitrogen content, plants may reduce the numbers of lateral roots while increasing the length of each lateral root (Walch-Liu et al., 2006; Postma et al., 2014). Such adjustments would confer a larger, broader root system, which is favorable for nitrogen acquisition. Therefore, growth modulation represents a strategy for plants to survive in changeable circumstances, especially stressful conditions.

The regulation of the third growth parameter, growth trajectory represents another important strategy of plant adaptation. When gravity constitutes the sole environmental factor of concern, the root grows in a straight and centripetally downward manner into the soil. However, in nature, due to heterogeneous soil environments (such as hard solid) most root doesn’t grow straitly (Lee et al., 2020). Apparently, modulation of root growth trajectory serves as a widely adopted and basic mechanism of plant adaption. Root growth trajectory has brought attentions from researchers and/or scientists (Cassab et al., 2013; Su et al., 2017; Lee et al., 2020). Considering that the existing terms used to describe root growth trajectory, such as waving and coiling, are mostly qualitative or semiquantitative (Mullen et al., 1998; Roy and Bassham, 2014). Grabov et al. (2005) established a set of parameters to estimate the wavy amplitude of root growth trajectory. Interestingly, they found that mutant lines (e.g., *thr1* and *axr2*) showing curved root also had alterations in the abilities of their roots to adapt to several environmental factors, including physical obstacles, light or gravity (Grabov et al., 2005; Sato et al., 2014). Therefore, flexibility of root growth trajectory/direction may boost (or strengthen) the plant ability to adapt to the changes in soil conditions.

Some types of growth trajectory show some typical characteristics. For example, the growth trajectory of a waving root exhibits a significantly sinusoidal growth pattern. However, less is known about the physiological relevance and the underlying molecular mechanisms of those curved growth trajectory without typical characteristics (unlike waving or coiling). Recently, it has been reported that the root growth trajectory could be regulated by several biotic/abiotic stresses, including high salinity, hydrogen peroxide, soil hypoxia and bacteria (Sun et al., 2008; Eysholdt-Derzso and Sauter, 2017; Zhou et al., 2018; Jimenez-Vazquez et al., 2020). For example, curvature of root growth trajectory was exacerbated with increasing the NaCl medium content (Sun et al., 2008). The authors further suggested that the curved phenotype might be associated with compromised auxin signaling (Sun et al., 2008). Moreover, hydrogen peroxide could also induce enhanced deviation to gravity vector by disrupting the membrane localization of the auxin efflux carriers PIN-FORMED 1 (PIN1) and PIN2 (Zhou et al., 2018). Therefore, curved root growth trajectory might uncover the coordination of the roots to adversities. Importantly, the phytohormone ABA plays an important role in the response to the abovementioned stress conditions (Neuman and Smit, 1991; Benech-Arnold et al., 2006; Veselov et al., 2008), and hydrogen peroxide has been suggested to be an intermediate of ABA signaling in the given phenotypes (Pei et al., 2000; Hung and Kao, 2004). This finding strongly implies that the stress-related hormone ABA serves as a mediator (in a concerted manner) between the stress conditions and the curved root growth trajectory/direction.

Here we report that the increased ABA level could elicit the wavy root growth trajectory. The phenotype elicited by the ABA resembles to that of roots agravitropism. However, the latter (gravitropism) appears not affected by ABA. We thus named or called this phenotype “agravitropism-like.” We revealed that the agravitropism-like phenotype resulted from a reduction in the total shootward auxin concentration. Furthermore, ABA mitigates the shootward auxin content through suppressing *PIN2* expression (an auxin efflux carrier), thus attenuating its membrane abundance. Such an attenuation of PIN2 abundance could also lead to agravitropism-like root. Overall, we present the regulatory effect of ABA on the root growth trajectory, and we further emphasized the role of ABA-auxin crosstalk in the context of root growth trajectory. These results may constitute a molecular network linking environmental factors to root growth trajectory.

## Materials and Methods

### Plant Material and Growth Conditions

The following mutant lines were used in this study: a double mutant *cyp707a1,3* generated by crossing *cyp707a1-1* (*At4g19230*) with *cyp707a3-1* (*At5g45340*) (Okamoto et al., 2006); The ABA signaling triple mutant, *afb2abf3abf4* (Yoshida et al., 2015). A single mutant line in *PIN2* (At5g57090), *pin2-1* (CS8056) (Qi and Zheng, 2013); The auxin influx carrier mutant, *aux1* (Marchant et al., 1999). the auxin receptor mutant line, *tir1-1* (CS3798; Ruegger et al., 1998), purchased from the Arabidopsis Biological Resource Center (SALK). Ecotype Colombia (Col-0) is the background of the five mutants: *cyp707a1,3, pin2-1*, *afb2abf3abf4*, *aux1* and *tir1-1.*

The following fluorescent marker lines were used: PIN2:PIN2-GFP (PIN2-GFP), GFP-tagged PIN2 protein in the Col-0 background (Birnbaum et al., 2003); DR5:VENUS, a synthetic auxin-responsive reporter line (Heisler et al., 2005); PIN1:PIN1-GFP GFP-tagged PIN1 protein in the Col-0 background (Benková et al., 2003). PIN2:PIN2-EosFP (PIN2-EosFP). PIN2-EosFP was generated following transformation process using the pCambia1300 backbone to insert PIN2:PIN2-EosFP in the plasmid (Dhonukshe et al., 2007).

PIN2-GFP/*cyp707a1,3* was generated by crossing PIN2-GFP with *cyp707a1,3*. To obtain PIN2-EosFP, PIN2-EosFP/*pin2-1* and PIN2-EosFP/*cyp707a1,3*, the pCambia1300-PIN2:PIN2-EosFP construct was introduced into the Col-0, *pin2-1* and *cyp7071,3* background, respectively. Among the T_3_ transgenic lines of PIN2-EosFP/*pin2-1*, one weak line and another strong one were chosen according to their *PIN2* expression levels (low and high, respectively) and tagged as W-rev-PIN2 and S-rev-PIN2. To express PIN2 specifically in the epidermis, PIN2-EosFP under the driver *GL2* (At1g79840) promoter (from −2184 to +25) was introduced into the *pin2-1* mutant. This epidermal PIN2 recovery line was named GL2:PIN2-EosFP in the current study.

Arabidopsis seeds were sterilized in 75% (V/V) ethanol for 5 min and sodium hypochloride for 15–30 min. Then, they were washed 5–6 times with sterilized deionized water and sown onto 1/2 MS medium (2.2 g l^–1^ MS salts, 1% sucrose, 10 g l^–1^ bacto-agar pH 5.8 with 1 M KOH). Afterward, they were incubated for 2-day at 4°C for stratification and dormancy removal, thereafter Petri dishes containing seeds were transferred to a growth chamber under controlled conditions (temperature, relative humidity, RH and photoperiod). These parameters were set as follows: 22°C, 80% (RH) and 16/8-h light/dark.

### Measurement of VGI and Gravitropic Bending Rate

To measure the root VGI following ABA treatment, 5-day-old seedlings geminated on 1/2 MS were transferred onto 1/2 MS medium either with or without ABA. Three days after transfer, the seedlings were photographed for subsequent VGI measurement. The root VGI was measured using ImageJ software according to the photos (version Java 1.8.0_112)^1^. For the measurements of root gravitropic bending rates, 5-day-old Col-0 seedlings were transferred onto the corresponding media (with/without ABA addition) one day before gravistimulation. Roots were placed horizontally for gravistimulation. Photos were obtained at 3, 6, and 9 h after gravistimulation. Root gravitropic bending rates were assessed according to the images obtained at each time point (3, 6, and 9h) using an ImageJ software.

**FIGURE 1:**
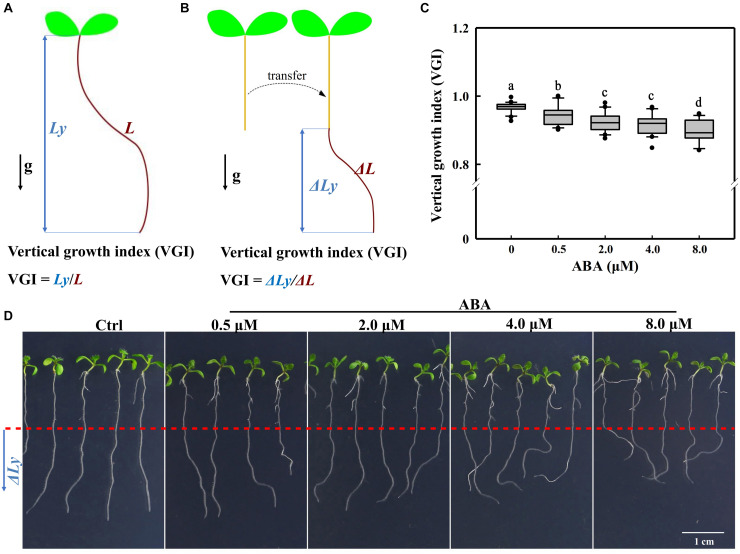
Definitions of the VGI and root VGI in the presence of different ABA concentrations. **(A)** A schematic diagram defining VGI before the transfer operation. *Ly*: vertical primary root length (root depth; the blue double-headed arrow); *L*: total root length (the brown curved line). VGI equals the ratio of *Ly* to *L*: VGI = *Ly/L*. **(B)** A simple schematic diagram illustrating the VGI of seedlings that have undergone the transfer operation. Seedlings grown on 1/2 MS medium, then transferred on a new medium (with ABA). Δ*Ly* and Δ*L* of the root were measured 3-day after transfer. Δ*Ly*: increased root depth after transfer (blue double-headed arrow); Δ*L*: increased root length after transfer (brown curve). VGI denotes the ratio of Δ*Ly* to Δ*L*: VGI = Δ*Ly*/Δ*L.* **(C)** Box plot showing the root VGI in the presence of different ABA concentrations (0.5, 2, 4, and 8 μM). Five-day old seedlings before being grown on 1/2 MS medium were transferred onto 1/2 MS media containing different ABA concentrations. As the control (Ctrl), seedlings were transferred onto the common 1/2 MS medium without any additions. At 3-day after transfer, the seedlings were photographed and then VGI measured. *n* > 30. **(D)** Representative images of Col-0 seedlings in the absence (Ctrl) or presence of different ABA concentrations as mentioned in panel **(C)**. The bottom root parts blow the red-dashed line represent the elongated roots after transfer.

**FIGURE 2:**
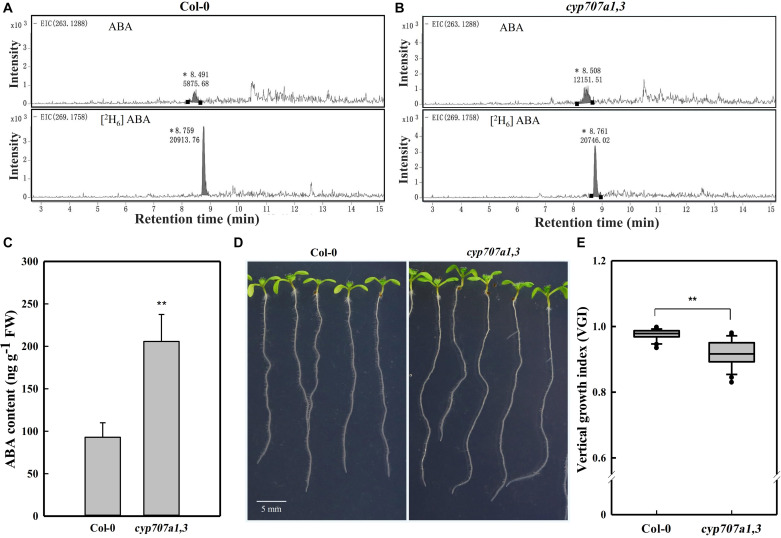
HPLC-MS dependent quantification of ABA contents in primary roots, the root VGI of WT (Col-0) and *cyp707a1,3*. **(A)** Extracted ion chromatograms (EICs) of ABA and its internal standard [^2^H_6_] ABA from extracts of 7-day-old Col-0 root tip. EICs of ABA (upper panel) and [^2^H_6_] ABA (bottom panel) were extracted from masses of 263.1288 ± 10 and 269.1758 ± 10 for ABA and [^2^H_6_] ABA, respectively. Integral peak of ABA (retention time: ∼8.49 min) and [^2^H_6_] ABA (retention time: ∼8.75 min) were measured for the subsequent evaluation of ABA content. **(B)** EICs of ABA and the internal standard [^2^H_6_] ABA from the *cyp707a1,3* double mutant extract. The integral peaks of ABA (retention time: ∼8.50 min) and [^2^H_6_] ABA (retention time: ∼ 8.76 min) were measured to qualify the ABA content in the root tip of *cyp707a1,3*. **(C)** The ABA content in the primary root tip of Col-0 and *cyp707a1,3*. ABA contents were evaluated according to the ratio of the integral peak of ABA and its respective standard [^2^H_6_] ABA. Each bar represents the mean of 3 biological samples and 3 technical replications ± SE. **(D)** Root VGI of Col-0 and *cyp707a1,3*. The images display 7-day-old seedlings grown on 1/2 MS medium. **(E)** The box plot presents the root VGI of the WT (Col-0) and double mutant *cyp707a1,3*. *n* > 30. * and ** represent *p* < 0.05 and *p* < 0.01 respectively.

For roots that have not undergo the transfer operation (Figures 2, 8 and Supplementary Figure 5), VGI is equal to the ratio of vertical root length to the total root length (Figure 1A), i.e., *Ly*/*L*. When seedlings were transferred onto a new medium for hormonal treatments (ABA and/or IAA), VGI represented the ratio of increased root depth (Δ*Ly*; Figure 1B, blue double-headed arrow) to the elongated (supplemental) root length (Δ*L*; Figure 1B, brown curve) after transfer, i.e., Δ*Ly*/Δ*L*.

### Plant Hormonal Treatments

For the VGI measurements under phytohormone (ABA and IAA) treatments, 5-day-old seedlings grown on 1/2 MS medium were transferred onto various new media containing the relevant plant hormones (ABA and/or IAA). Three d after transfer, the seedlings were photographed for subsequent VGI determination. When seedlings are prepared for confocal microscopy, the hormonal treatment endured only 1 d, after which the samples were loaded for observation using confocal microscope.

### Quantification of ABA Content

ABA extraction from the root tip of the WT and the double mutant *cyp707a1,3* was conducted following the method of Pan et al. (2010). Notably, seeds were mounted very tightly on the plate for easy sampling. Totally 50 mg of root tip (fresh weight; ∼4 mm in length) collected from either WT or *cyp707a1,3* was ground in liquid nitrogen. Subsequently, 20 ng of [^2^H_6_] ABA (Icon Isotopes, cat. no. ID1001; 0.5 ng μl^–1^ dissolved in menthol) was added as the internal standard. Subsequently, 500 μl extraction buffer was added to each sample. The extraction buffer contains 2-propanol/H_2_O/HCl (2:1:0.002 v/v/v). The samples were mixed well using a shaker operating at 100 rpm under 4°C for 30 min. Then, 1 ml dichloromethane was added and the shaking was continued for an additional 30 min under the same conditions. Afterward, the samples were centrifuged at 13,000 rpm at 4°C for 5 min. Then, ∼900 μl liquid of the lower phase was gently transferred and the upper phase discarded. A gentle handling is recommended to avoid disruption of the pellet (solid phase) located between the two liquid phases (lower and upper). Subsequently, the samples (∼900 μl) were concentrated using nitrogen flow without complete drying. The samples were then dissolved in 0.1 ml methanol and stored at −80°C for a later HPLC analysis. The ABA content in each sample was determined on an Agilent liquid chromatography-tandem mass spectrometry device system equipped with a ZORBAX Eclipse XDB-C18 column as described previously (Yano et al., 2009; Weng et al., 2015).

### RNA Isolation and Quantitative Real-Time Polymerase Chain Reaction (qRT-PCR)

Total RNA from the root tip of Col-0, single mutant (*pin2-1*), double mutant (*cyp707a1,3*) and transgenic lines (W-rev-PIN2 and S-rev-PIN2) was extracted following the manufacturer’s instructions supplied with the total RNA isolation kit (RNeasy, QIAGENE, No. 74904). One hundred nanograms of the isolated RNA from each sample was used to reverse transcribe (synthesis) the first-strand cDNA using ReverTra Ace qPCR RT MASTER MIX (with gDNA remover; TOYOBO, No. FSQ-301). The qRT-PCR was conducted using Real-time PCR Master Mix (TOYOBO, No. QPK-101) with 3 biological replicates for each sample. The parameters used for qRT-PCR cycling were as follows: 95°C for 10 s, 55°C for 20 s, 72°C for 20 s. The primer sequences, GCCGAGAGCTTCTAGCTTTAA (forward) and CGCCGTAGCTATTAGTGTAACC (reverse), were used for the amplification of *PIN2.* The primer sequences, TCGGTCGTTGTATTGTGCTTT (forward) and CCAGATGCTCATTACACACTCA (reverse), were used for the amplification of *RAB18* (At5g66640). For the reference gene *Actin2*, GCACCCTGTTCTTCTTACCG (forward) and AACCCTCGTAGATTGGCACA (reverse) were used. The primer sequences AACTTTGGTGGTTTGTGTTTTGG (forward) and TCGACTTGTCATTAGAAAGAAAGAGATAA (reverse) were used for the amplification of another reference, *UBQ10*. Because the results using *Actin2* or *UBQ10* as the reference were identique, we used *Actin2* to normalize *PIN2* expression. The relative *PIN2* expression compared with *Actin2* was calculated using the 2^–ΔΔ*CT*^ method as described previously (Livak and Schmittgen, 2001).

### Auxin Immunofluorescence

Root tips obtained from 5-day-old Col-0 and *cyp707a1,3* seedlings were prefixed at 4°C for 30 min in cross-linking buffer that could cross-link the carboxyl group of free IAA to structural proteins and preserve its antigenicity (Márquez-López et al., 2018). This cross-linking buffer consists of 3% *N*-(3-dimethylaminopropyl)-*N*′-ethylcarbodiimide hydrochloride (EDAC; Sigma, No. 03449) in 1 × phosphate-buffered saline (1 × PBS). After prefixation, the samples were fixed in FAA solution (formaldehyde alcohol acetic acid; 50% ethanol, 5% acetic acid, 37% formaldehyde; 18:1:1) overnight at 4°C. The samples were then washed 3 times with 1 × PBS for 5 min each. Thereafter, the cell walls were digested with 1% Macerozyme R-10 and 1% Driselase (dissolved in 1 × PBS) for 18 min at 37°C. Subsequent membrane permeabilization was conducted after digestion by submerging the samples in the permeabilization solution (3% IGEPAL CA-630 and 10% DMSO in 1 × PBS) for 20 min at 37°C. The samples were then washed 5 times with 1 × PBS for 5 min each. Afterward, the samples were submerged in blocking buffer (3% BSA dissolved in 1 × PBS) for 90 min at room temperature (20-22°C). Thereafter, they (samples) were incubated overnight with mouse anti-IAA monoclonal antibody (1:200 dissolved in 3% BSA in PBS); Sigma) at 4°C and subsequently washed 3 times with 1 × PBS. Finally, the samples were incubated with Alexa Fluor 488 goat anti-mouse IgG secondary antibody (1:100; Abcam) for 2 h at 37°C followed by 3 washes (5 min each) with 1 × PBS. For examination, the root tips were mounted onto glass slides for confocal microscopy observations. The immunofluorescence protocol was performed as documented previously with slight modifications (Pasternak et al., 2015; Márquez-López et al., 2018).

### Confocal Microscopy and Fluorescence Intensity Measurements

**FIGURE 3:**
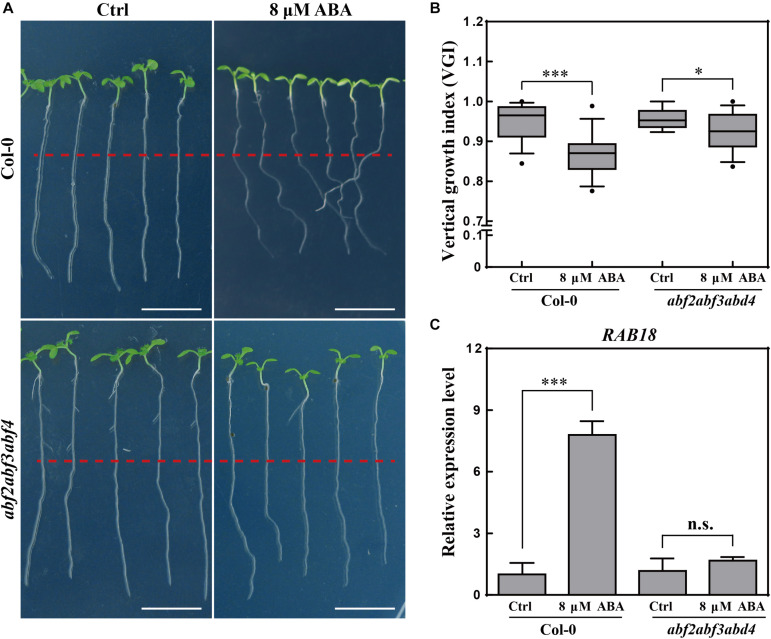
Root VGI of Col-0 and *abf2abf3abf4* in response to ABA treatment. **(A)** Representative images of Col-0 and *abf2abf3abf4* seedlings for control and following ABA treatment. 5-day-old seedlings were treated with 8 μM ABA; the photographs were taken 3-day after transfer. White bar represents a scaling bar of 1 cm. *n* > 30. **(B)** Root VGI of Col-0 and *abf2abf3abf4* seedlings for control and following ABA treatment. Each bar represents the mean of 30 independent measurements ± SE. **(C)** Relative expression level of *RAB18* in roots of Col-0 and *abf2abf3abf4* with and without ABA treatment. Each bar represents the mean of 3 biological samples and 3 technical replicates ± SE. * and *** represent *p* < 0.05 and *p* < 0.001 respectively.

**FIGURE 4:**
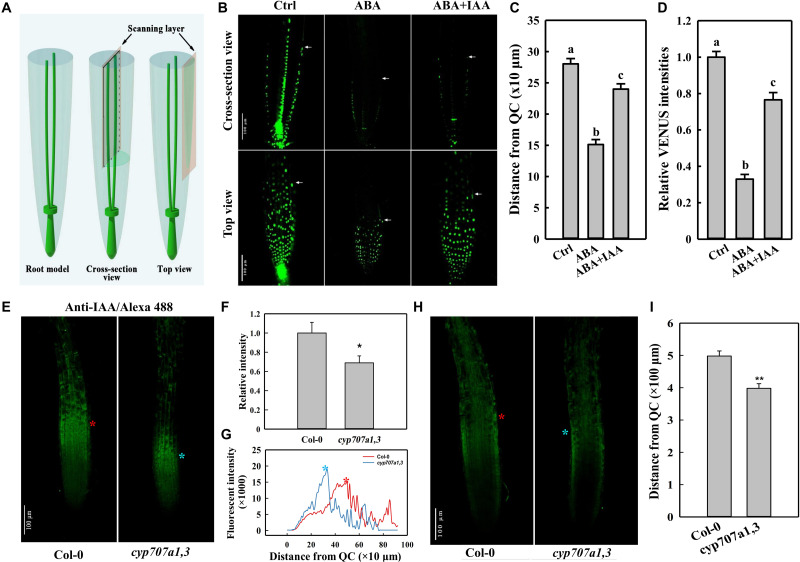
DR5:VENUS signal in the root tip following different treatments, and immunofluorescence of IAA in the root tip of Col-0 and *cyp707a1,3*. **(A)** Schematic diagram of the scanning layers of the cross-section (middle panel) and top views (right panel). The cross-section view refers to the image generated by scanning through the middle of the root tip (middle panel). The top view was generated by scanning through the surface of a root. **(B)** Cross-section and top views of DR5:VENUS signal in the control root tip and those treated with plant hormones. Five-day-old DR5:VENUS seedling were transferred onto control (1/2 MS without hormonal addition), ABA (1/2 MS with 8 μM ABA) or ABA + IAA (1/2 MS with 8 μM ABA together with 5 nM IAA) media. Following a single day of treatment, the seedlings were loaded onto a confocal microscope for imaging. White arrowheads indicate the ends of DR5:VENUS signals. White triangles indicate PIN2-GFP signals in cells of EZ. **(C)** Coverage of SAT as reflected by the distance from the end of the epidermal DR5:VENUS signal (panel b, white arrowheads) to the QC. Measurements were conducted according to the cross-section view in the panel **(B)**. **(D)** Relative epidermal DR5:VENUS intensities following the same treatments as in panel **(B)**. Fluorescence intensities were normalized to the control. Measurements were conducted according to the top view in panel **(B)**. **(E)** Top view of the anti-IAA/Alexa Fluor 488 (IAA/Alexa 488) signal in Col-0 and *cyp707a1,3*. Primary roots (PRs) of 5-day-old Col-0 and *cyp707a1,3* seedlings were immunodetected using anti-IAA/Alexa Fluor 488. Fluorescence intensities were normalized to Col-0. **(F)** Total intensity of IAA/Alexa 488 in the root tip of Col-0 and *cyp707a1,3*. **(G)** Vertical profiles of IAA/Alexa 488 signal in the epidermis. Line profiles were generated from the epidermal IAA/Alexa 488 signal as shown in panel **(E)**, according to its fluorescence intensity along the vertical direction of the root. **(H)** Cross-section view of IAA/Alexa 488 in the root tip. The stars (red and blue) in this panel, as well as those in panels **(E,G)**, indicate the intensity peak of the IAA/Alexa 488 signal in Col-0 (red) and *cyp707a1,3* (blue). *n* = 15. **(I)** Coverage of acropetal auxin flux (AAF) evaluated by the distance from the intensity peak of the epidermal IAA/Alexa 488 signal [panel **(H)**, red and blue stars] to QC. Measurements were performed according to the cross-section view. For the histograms shown in panels **(C,D)**, each bar data represents the mean of 30 independent measurements ± SE. * and ** represent *p* < 0.05 and *p* < 0.01 respectively.

Root tips were mounted on glass slide for confocal microscopy. In this study, an Olympus FV1000^2^ was used for confocal microscopy. Unless otherwise specified, the parameters for confocal imaging between different trails or time points within an independent experiment were completely the same. For DR5:VENUS (YFP), we used the 515-nm band of an argon laser for excitation. A 488-nm wavelength was used for GFP; 559 nm was used for propidium iodide (PI) excitation. The 2 image types for the DR5:VENUS signal in Figure 3 were obtained by scanning through different sections of roots. The cross-section view was generated by scanning through the middle of a root (Figure 4A, middle panel). The top view was generated by scanning through the top surface of the root tip (Figure 4A, right panel). The photoconversion operation shown in Figure 9 was performed by exposing the whole root tip to 405-nm wavelength radiation for 30 s. Hence, 5-day-old seedlings were used in these experiments.

The raw data for fluorescent images generated using an Olympus FV1000 confocal microscope were manipulated using Image-Pro Plus software (v6.0), in which membranes were outlined automatically with the same threshold gray value. Average gray values were used to represent the fluorescence intensities, which were finally normalized to their respective control (explained in detail in the figure legend).

### Statistical Data Analysis

The data were collected in Microsoft Excel 2019. We performed ANOVA followed by Tukey’s pairwise comparisons at a *P*-value <0.05 for the following analyses: ABA content in Col-0 and *cyp707a1,3* (Figure 2C); root VGI of Col-0 and *cyp707a1,3* (Figure 2E); *RAB18* expression in Col-0 and *abf2abf3abf4* (Figure 3B); VGI of Col-0 and *abf2abf3abf4* in control or following ABA treatment (Figure 3C). IAA/Alexa 488 intensity in Col-0 and *cyp707a1,3* (Figure 4F); IAA/Alexa 488 signal coverage in the epidermis of Col-0 and *cyp707a1,3* (Figure 4I). The statistical analysis for the other parameters, we performed ANOVA followed by Tukey’s multiple comparison tests at a *P*-value <0.05.

Concerning the box plots, the central vertical bar in the box represents the median, and the box represents the interquartile range. Two plot whiskers span 1.5 times the interquartile range of the distribution. The dots outside of whiskers denote outliers.

## Results

### ABA Elicits an Agravitropism-Like Root Phenotype

To investigate whether ABA could control the root growth trajectory, we first transferred 5-day-old WT (Col-0) seedlings onto 1/2 Murashige and Skoog (MS) medium containing different concentrations of ABA (Figure 1). We implemented a transfer operation due to the inhibitory effect of ABA on germination (Ackerson, 1984; Finkelstein et al., 2002; Liu et al., 2013). We found that ABA could elicit an agravitropism-like phenotype, which presents wavy growth trajectory/direction (Figure 1). To quantify the wavy amplitude of root growth trajectory, we adopted a reported parameter, the vertical root growth index (VGI; Figure 1A; Grabov et al., 2005). To avoid the inaccuracy that can be introduced by the transfer operation, we provided an additional definition to VGI for the transferred seedlings. Thus, VGI of the transferred seedling equals the ratio of increased root depth (Δ*Ly*; the blue double-headed arrow in Figure 1B) to the elongated root length (Δ*Ly*; the curved line in Figure 1B) after transfer, i.e., Δ*Ly*/Δ*L* (Figure 1B). Briefly, a lower VGI represents an exaggerated curvature or wavy amplitude of root growth trajectory. Hence, we transferred 5-day-old seedlings onto mediums containing 0.5–8 μM ABA. Notably, the used ABA concentrations that cover relatively a broader range, which may reflect various environmental conditions. Using various ABA concentrations, we found that ABA significantly reduces the root VGI (Figures 1C,D), indicating that the root growth trajectory could somehow be regulated by ABA.

The addition of ABA may affect the physicochemical properties of the medium, and thus the agravitropism-like phenotype could result from the altered surface characteristics of the medium. To exclude this possibility, we focused to determine whether the agravitropism-like phenotype could be manifested by an endogenous ABA accumulation. Herein, we investigated the VGI in the double ABA-degradation mutant, *cyp707a1cyp707a3* (*cyp707a1,3* hereafter), which is known by its higher ABA content in the dry seeds by approximately 20 folds if compared to WT (Okamoto et al., 2006). Since both *CYP707A1* and *CYP707A3* are mainly expressing in the roots (Winter et al., 2007) and our current work focuses on root growth, we accordingly expect an increase in the endogenous ABA content in the primary root of the *cyp707a1,3* double mutant. To confirm this finding, we quantified the ABA content in the primary root of *cyp707a1,3* using high-performance liquid chromatography (Figures 2A,B), and verified corresponding intensity peak via the tandem mass spectrometry (Supplementary Figure 1). We detected an approximately 2 folds higher ABA content in the primary root tip of *cyp707a1,3* compared to the WT (Figures 2A,C). In line with the results obtained for exogenous ABA application, the VGI of *cyp707a1,3* was significantly lower than that in the WT (*p* < 0.01; Figures 2D,E). Therefore, instead of the alteration of surface characteristics of the medium, ABA accumulation represents the main reason for the agravitropism-like phenotype (Figures 1, 2).

To better understand the relationship between ABA and the agravitropism-like phenotype, a triple mutant line in three transcription factors of ABA signaling, the *abf2abf3abf4* (Yoshida et al., 2015) was tested for its response to ABA treatments. The VGI of *abf2abf3abf4* was far less sensitive upon ABA application (Figures 3A,B). To assess the ABA signaling in *abf2abf3abf4*, the expressions of a widely used ABA responsive gene *RAB18* (Yoshida et al., 2015) were quantified in WT and the mutant. Hence, the exogenous ABA (8 μM) application considerably increases the *RAB18* expression in WT, meanwhile its (*RAB18*) expression level in the *abf2abf3abf4* is found to be less sensitive to ABA treatment (Figure 3C). Therefore, the ABA-elicited agravitropism-like phenotype is impaired in the ABA signaling mutant. Otherwise, ABA affects the root growth trajectory through partially the ABRE BINDING FACTORs (ABFs) pathway.

### ABA Attenuates Shootward Auxin Content in the Root Tip

Auxin is transported from the root cap back toward the root elongation zone (EZ) through an auxin efflux carrier PIN2 (Friml, 2003; Kramer and Bennett, 2006). The auxin transport in this direction was earlier termed shootward auxin transport (SAT; Friml, 2003; Kramer and Bennett, 2006). Certain SAT mutants (e.g., *pin2*) show a curved root growth trajectory and gravitropic growth pattern (Bennett et al., 1996; Chen et al., 1998; Müller et al., 1998; Marchant et al., 1999). Thus, the agravitropism-like phenotype induced by ABA likely also to be resulted from the alterations in SAT. Thus, we used the auxin reporter line DR5:VENUS to determine whether SAT could be influenced by ABA. Based on the cross-section view, which presents the clear signal ends in the epidermis (Figures 4A,B), we could observe a significantly reduced coverage of the DR5:VENUS signal in the epidermis upon ABA treatment (Figures 4B,C). Moreover, according to the top view, which provides an accurate information regarding fluorescence intensity (Figures 4A,B), the epidermal DR5:VENUS intensity was strongly reduced following ABA application (Figures 4B,D). Hence, the Reduction in both the signal intensity and coverage implies the mitigation in the shootward auxin content. It’s worthy to note that auxin is not accumulated obviously in the root caps or QC although the impairment of its SAT. This results from a decreased rootward auxin transport as indicated by the reduced PIN1 abundance (Supplementary Figure 2), which is involved in the auxin transport from the up-ground source to QC and root caps.

To verify whether the endogenous ABA accumulation could cause the same attenuation in auxin content, we investigated the auxin distribution in the root tip of *cyp707a1,3* and WT (Col-0) via immunofluorescence (Figures 4E,H). Using anti-IAA/Alexa Fluor 488 to detect the auxin in the root tip, we thus demonstrated that the total anti-IAA/Alexa Fluor 488 (IAA/Alexa 488) intensity in the epidermis of *cyp707a1,3* was significantly reduced compared to the WT (Figures 4E,F). As reflected by the vertical profile (signal intensity distribution along the vertical direction) of the top-view signal (Figure 4G), the IAA/Alexa 488 in *cyp707a1,3* appears to reach its intensity peak much earlier (closer to quiescent centre; QC) than WT (Figures 4E,G, red and blue stars). In addition, the immunofluorescence signals in the EZ of the *cyp707a1,3* double mutant were almost undetectable; however, the signal in the same region of WT was significantly stronger (Figures 4E,G). As shown by both the cross-section and top views (Figures 4E–H; indicated by red and blue stars), the distance from the QC to the intensity peak of IAA/Alexa 488 was shorter in *cyp707a1,3* than in WT (Figure 4I). This indicates that the total auxin content of SAT was weakened in *cyp707a1,3*, since both the signal coverage and intensity were decreased (Figures 4E–I). Thus, both the exogenous and endogenous enhancements in ABA level could reduce the shootward auxin content.

**FIGURE 5:**
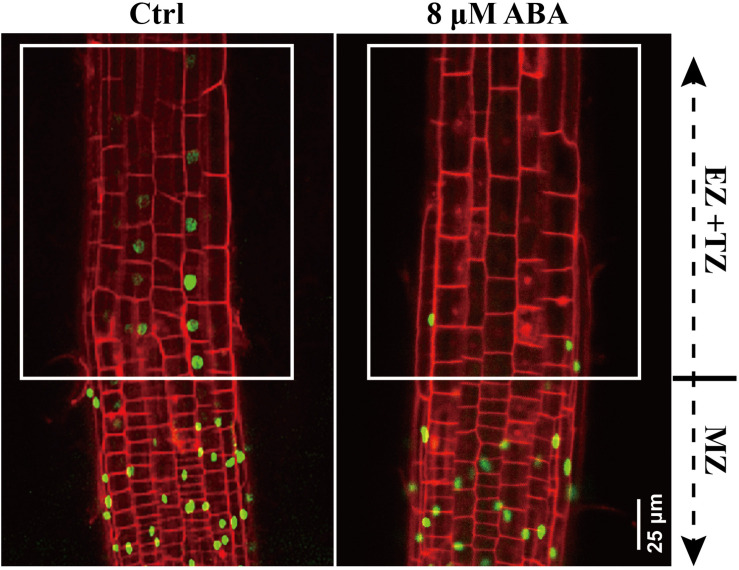
DR5:VENUS signaling in control and ABA-treated seedling. 5-day-old DR5:VENUS seedlings were treated with 8 μM ABA for 1-day. Roots were stained by PI and imaged by confocal microscope. MZ represents meristem zone, EZ and TZ represent the elongation zone (EZ) and transition zone (TZ), respectively. PI is the propidium iodide.

The physical distance between QC and the end of DR5:VENUS signal could be affected if the ABA application causes a shorter meristem (Supplementary Figure 2; indicated by white arrowheads). In this regard, we employed a PI staining to visualize auxin distribution pattern in different root zones. In WT roots, auxin considerably distributed from the meristem zone to the EZ (including transition zone; Figure 5). Following ABA application, DR5:VENUS signals were mainly restricted to meristem (Figure 5). Hence, it appears that ABA triggering a reduction in DR5:VENUS coverage is not only attributable to the reduced meristem size, but also to the impaired SAT. Since roots bend after transfer onto an ABA medium, we decide to examine the epidermal cell elongation in the bending site of roots treated with ABA. The cells in convex and concave root face do not elongate equally (Supplementary Figure 3). Hence, we speculate that certain auxin level is required to maintain the symmetric cell growth in these regions. In conclusion, ABA impaired SAT confers low auxin concentration in EZ. Deficiency in auxin may not insufficient to maintain the symmetrical growth of cells in this region, further results in the root bending.

Since we demonstrated that ABA could attenuate the shootward auxin content (Figures 4, 5) and considering the assumption that SAT correlates with the root growth trajectory (Sun et al., 2008), we speculate that the agravitropism-like phenotype caused by ABA was due to the mitigated auxin level of SAT. To confirm this hypothesis, we added 50 nM IAA together with 8 μM ABA to the medium. Compared to the ABA application alone, the DR5:VENUS intensity was increased in the epidermis following IAA application together with ABA (Figures 4B–D). This result suggests that the external application of IAA could partially compensate for the ABA-induced auxin diminution of SAT. Hence, we added IAA together with ABA to confirm whether the agravitropism-like phenotype could be rescued. As predicted, IAA greatly recovered the root VGI to the same level of the control (Supplementary Figure 4). However, in the auxin receptor mutant *tir1-1*, the compensatory effect was completely lost (Supplementary Figure 4). Accordingly, ABA-reduced root VGI could be rescued by the recovery of the shootward auxin level. Thus, ABA-attenuated the shootward auxin content could influence the root growth trajectory.

### PIN2 Is Required for the ABA-Modulated Root Growth Trajectory

**FIGURE 6:**
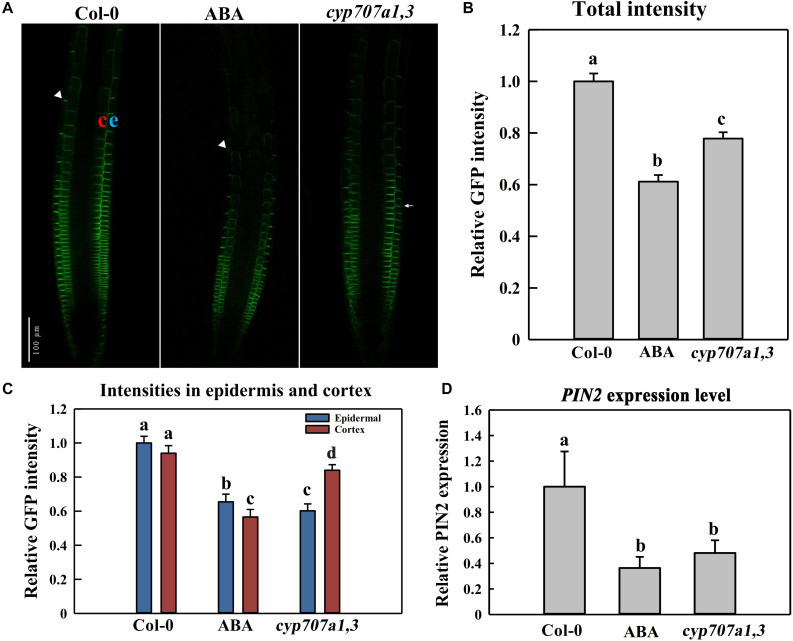
PIN2 abundance and distribution in the control Col-0, ABA-treated WT (Col-0) and *cyp707a1,3* double mutant. **(A)** Representative confocal images of PIN2-GFP in Col-0, ABA-treated WT and *cyp707a1,3*. For ABA-treated WT (middle panel), 5-day-old PIN2-GFP/Col-0 seedlings were treated with 8 μM ABA for an additional d. For Col-0 and *cyp707a1,3* (left and right panels, respectively), 5-day-old seedlings were transferred onto 1/2 MS medium 1-day before confocal microscopy observations. **(B)** Relative total intensity of PIN2-GFP in different lines or treatments was as described in panel **(A)**. Total PIN2-GFP intensities refer to the GFP intensity on the membrane (outlined automatically with the same threshold) and were normalized to PIN2-GFP/Col-0. **(C)** PIN2-GFP distribution in the epidermis and cortex of different lines or treatments was as described in panel **(A)**. Intensities in each layer of all trials refer to the membrane intensities and were normalized to the epidermal PIN2-GFP intensity of PIN2-GFP/Col-0. **(D)** Relative *PIN2* expression level in the root of the control Col-0, ABA-treated Col-0 and *cyp707a1,3*. Col-0 and ABA represent 5-day-old Col-0 seedlings treated without (Col-0) or with ABA. *cyp707a1,3* was transferred onto 1/2 MS-free medium. Following 1-day treatments in the new medium, *PIN2* expression level in the root tip was analyzed by qRT-PCR. The expression level was normalized to WT (Col-0). Each bar represents the mean of 3 biological samples and 3 technical replicates. For the histograms of panels **(B,C)**, each bar represents the mean of 30 independent measurements ± SE. For panels **(B–D)**, the adjacent letters denote the significance levels.

We mentioned in the precedent paragraph that ABA causes the agravitropism-like phenotype by reducing the auxin content of SAT. It is well documented that SAT is propelled (or driven) by an auxin efflux carrier, PIN2 (Müller et al., 1998; Shin et al., 2005). Hence, ABA-induced attenuation of the SAT very likely resulted from the reduced PIN2 abundance. To verify that, we investigated the PIN2 abundance and distribution in the root tip following ABA treatment. Hence, using a reporter line, PIN2:PIN2-GFP (PIN2-GFP, Thereafter), which expresses GFP-tagged PIN2 in WT (Col-0 background), we observed a significantly reduced PIN2 abundance upon 8 μM ABA treatment (Figures 6A,B). We also crossed PIN2-GFP with *cyp707a1,3* (double mutant) to introduce PIN2-GFP into *cyp707a1,3*, generating thus the PIN2-GFP/*cyp707a1,3* line. In line with the results showing the ABA attenuation of the auxin content of SAT (Figure 4), the total membrane PIN2 intensity in PIN2-GFP/*cyp707a1,3* was also weaker than in the WT (especially in EZ; Figures 6A,B, white triangles). Therefore, the accumulated ABA could reduce the membrane PIN2 abundance. The PIN2 is located in the epidermal membrane situated in the outermost layer of the root (Figure 6A; left panel, indicated by letter e) and the cortex layer directly adjacent to the epidermis (Figure 6A; left panel, depicted by the latter c). In this regard, we found that the decrease in PIN2-GFP intensity of *cyp707a1,3* was mainly attributed to its attenuation in the epidermis (∼40% reduction compared with Col-0) rather than the cortex (∼15% reduction; Figure 6C).

We also analyzed the PIN2 expression level in response to ABA application or in *cyp707a1,3* mutant. Thus, the PIN2 expression level upon either ABA application or in *cyp707a1,3* was deceased compared to the WT (Figure 6D). Hence, the reduced PIN2 abundance is partially due to the suppression of *PIN2* transcription. Besides, the *PIN2* expression level between ABA application and *cyp707a1,3* didn’t show any significant difference; however, the PIN2 intensity in *cyp707a1,3* is stronger than for the ABA treatment. Although both ABA application and mutant line generate higher ABA concentration, the ABA content in *cyp707a1,3* appears more accurately controlled, because of the spatial and temporal pattern of CYP707A1 and CYP707A3. For example, the subcellular zonation of these two enzymes (CYP707A1 and CYP707A3) might result in the missing of ABA induced post-transcriptional regulation of PIN2 in *cyp707a1,3*. In the scenario of ABA application, the ABA level increases ubiquitously and therefore inhibits the *PIN2* translation at the post-transcriptional level. Altogether, although *PIN2* expression level in ABA-treated root and *cyp707a1,3* seems equal (Figure 6D), the differences in post-transcriptional level may confer differences in PIN2 abundance.

**FIGURE 7:**
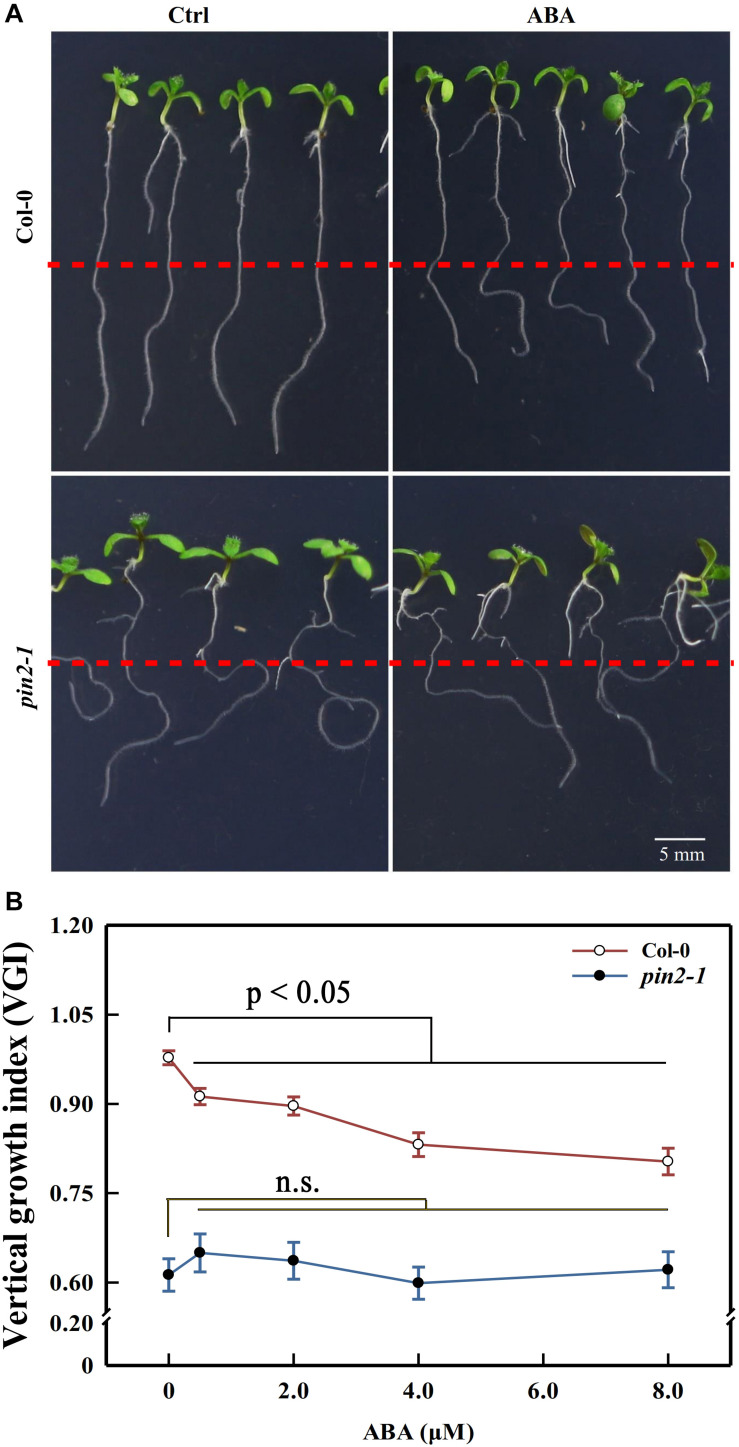
Primary root VGI of Col-0 and *pin2-1* in control and ABA-treated seedlings. **(A)** Seedlings of Col-0 (upper panels) and *pin2-1* (bottom panels) for control and ABA-treated seedlings. Five-d old Col-0 and *pin2-1* seedlings were transferred onto 1/2 MS medium without (Ctrl) or with 8 μM ABA. The root VGI was measured 3-day after transfer. **(B)** Root VGI of Col-0 and *pin2-1* evaluated on seedlings endured the same treatments described in panel **(A)**. Each data point is the average of 30 independent measurements obtained for 30 different seedlings ± SE.

Although we showed above that the attenuation in shootward auxin content could lead to the agravitropism-like root, it is possible that the reduction in membrane PIN2 abundance could also trigger the same phenotype. Moreover, it remains unclear whether ABA modulated the root growth trajectory through regulating the PIN2 abundance or via another unknown process. To decipher these possibilities, we recovered *PIN2* in *pin2-1* by introducing PIN2-EosFP into the mutant, and we obtained one strong line and another weak recovery line according to the *PIN2* relative expression level. The EosFP-tag conjugated to the PIN2 protein is a fluorescent protein (property of EosFP will be discussed latter; Lippincott-Schwartz and Patterson, 2008; Bourgeois et al., 2012). The weak allele (W-rev-PIN2) possesses a much higher expression level than *pin2-1*. However, its *PIN2* expression level represents only 20% of that in WT (Supplementary Figure 5A). Correspondingly, its VGI was higher than in *pin2-1* but lower than that in WT (Supplementary Figures 5B,C). The strong recovery line (S-rev-PIN2) shows a similar expression level to WT and close VGI values. Therefore, the agravitropism-like phenotype positively correlates with the *PIN2* expression level, signifying the involvement of PIN2 in controlling the root growth trajectory. We then assessed the VGI of the *pin2-1* mutant in the presence of different ABA concentrations. Compared with WT, in which VGI was decreased by ABA, VGI of *pin2-1* was completely insensitive to ABA treatment and was significantly reduced versus WT with or without ABA (Figure 7). Therefore, PIN2 was indispensable (plays a pivotal role) for the process of ABA root growth trajectory regulation. Alternately, ABA elicits the agravitropism-like phenotype by modulating the PIN2 abundance. Together with our above conclusion stating that ABA attenuates the SAT by decreasing the PIN2 abundance (Figure 6), these data support the deduction that ABA leads to the reduction of membrane PIN2 abundance, thereby attenuating the shootward auxin content and, consequently, the wavy root growth trajectory.

The growth trajectory of a similar mutant line *aux1* was also tested for its response to ABA. The AUX1 is an auxin influx carrier which allows the entry of extracellular auxin into intracellular space, and is also involved in the auxin transport from root caps to the EZ (Bennett et al., 1996). Surprisingly, unlike *pin2-1*, the VGI of *aux1* is still sensitive to ABA application (Supplementary Figure 6). The root of *aux1* even forms a coil upon ABA treatment. This might be due to the different auxin distribution between *aux1* and *pin2-1* (Swarup et al., 2001; Liu et al., 2018), although they are both involved in the SAT. Moreover, this may indicate that ABA regulates the root growth trajectory specifically through PIN2. Somehow, *aux1* also exhibits a reduced VGI if not taking in consideration the ABA. Thus, phenotype of *aux1* may ultimately correlates the impaired SAT with the agravitropism-like phenotype.

**FIGURE 8:**
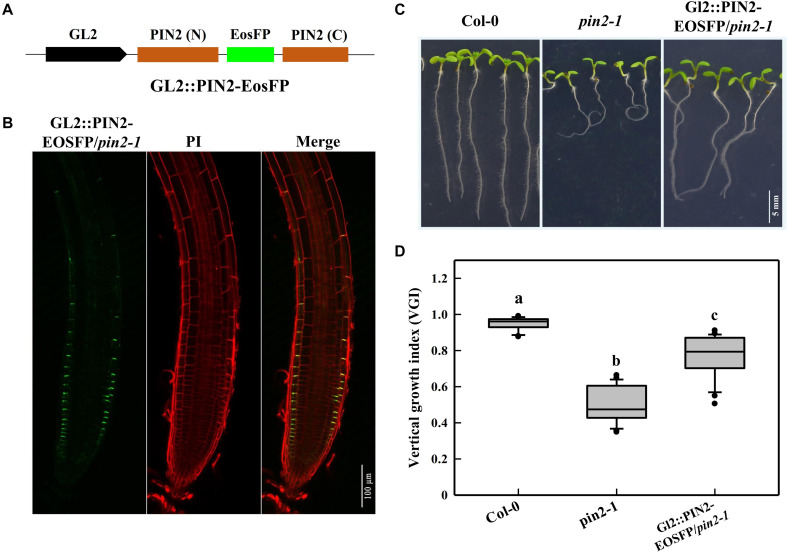
Root VGI of the Col-0, *pin2-1* and a partial recovery line GL2:PIN2-EosFP/*pin2-1*. **(A)** Schematic map of the GL2:PIN2-EosFP construct. GL2 represents the promoter of *GL2*, which is expressed specifically in the root epidermis. The fluorescent tag EosFP was inserted between the N and C-terminus of *PIN2* CDS. The construct was introduced into *pin2-1* mutant lines to generate a partial recovery line, GL2:PIN2-EosFP/*pin2-1*. **(B)** *PIN2* expression pattern in GL2: PIN2-EosFP/*pin2-1*. **(C)** Representative seedlings of Col-0, *pin2-1* and GL2:PIN2-EosFP/*pin2-1*. Seven-d-old seedlings are shown in the images. **(D)** The box plot shows the root VGI of Col-0, *pin2-1* and GL2:PIN2-EosFP/*pin2-1*. *n* > 30 for each bar.

We mentioned above that the decrease in PIN2 abundance of *cyp707a1,3* was mainly ascribed to its reduction in the epidermis rather than the cortex (Figure 6C). Thus, it was necessary to unravel whether the reduction in epidermal *PIN2* could lead to a wavy growth trajectory. For this purpose, we expressed the PIN2-EosFP under the driven GL2 promotor in the *pin2-1* mutant (Figure 8A). Because the GL2 promotor specifically expresses in the root epidermis (Lee and Schiefelbein, 1999), we obtained a partial PIN2 recovery line in which only epidermal PIN2 was expressed. We tagged this line GL2:PIN2-EosFP. Through confocal detection of the EosFP signal in GL2:PIN2-EosFP, we found that PIN2 was successfully expressed in the epidermis of this partial recovery line (Figure 8B). As expected, the VGI of GL2:PIN2-EosFP was significantly higher than in *pin2-1* mutant (Figures 8C,D). Thus, the epidermal PIN2 is required for controlling the root growth trajectory, and the reduction in the epidermal PIN2 abundance of *cyp707a1,3* could indeed result in a reduced VGI.

### ABA Reduces PIN2 Abundance Mainly Through Inhibiting Its *de novo* Synthesis Rather Than Accelerating Its Degradation

**FIGURE 9:**
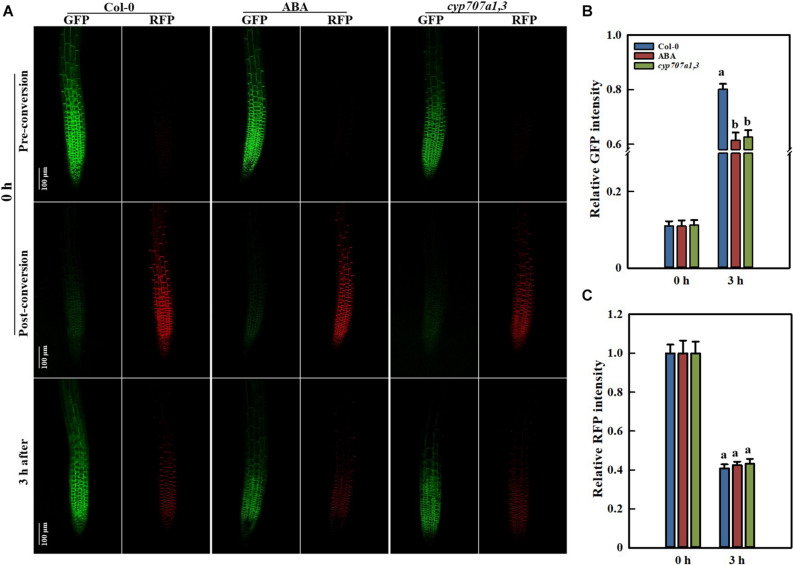
PIN2 synthesis and degradation in Control Col-0, ABA-treated Col-0 and the *cyp707a1,3* double mutant as revealed by the photoconversion of PIN2-EosFP. **(A)** Representative confocal images of PIN2-EosFP (including GFP and RFP) in Col-0, ABA-treated Col-0 and *cyp707a1,3*. The 6 upper panels show the pre-conversion images of PIN2-EosFP (GFP state) that was not yet converted into RFP. After a 30-s exposure to 405-nm irradiation, EosFP was converted to the post-conversion state in which most PIN2-EosFP was in the RFP state (middle panels). Three hours after conversion, the dynamics of the GFP and RFP signals (bottom panels) were measured to evaluate the rate of PIN2 synthesis and degradation. Five-d-old seedlings were used. After photoconversion, the PIN2-EosFP/Col-0 seedlings were directly transferred onto 1/2 MS medium with or without 8 μM ABA, and they were labeled Col-0 (left panels column) and ABA (middle panels column). For the PIN2-EosFP/*cyp707a1,3* seedlings were also transferred onto 1/2 MS after photoconversion (right panels column). **(B)** Increasing speed of GFP within 3 h. Col-0, ABA and *cyp707a1,3* seedlings were treated as described in panel **(A)**. Relative intensities of Col-0, ABA and *cyp707a1,3* refer to the membrane intensities normalized to their original GFP intensities (pre-conversion). **(C)** Decreasing rate of RFP within 3 h. Same figure legend as in panel **(B)**. For the histograms of panels **(B,C)**, each bar is the average of 30 independent measurements made on different roots ± SE. The relative intensities of Col-0, ABA and *cyp707a1,3* represent the membrane intensities normalized to their original RFP intensities (post-conversion).

The protein abundance is mainly determined by its degradation and *de novo* synthesis. A reduction in the PIN2 abundance might result from either its decreased synthesis or accelerated degradation. To characterize the way by which ABA reduces the root PIN2 abundance, we utilized the photoconversion fluorescent tag EosFP to segregate protein synthesis and degradation pathways. Due to the particularity of the photo-convertible material, once we implemented the photoconversion operation, the PIN2 protein already present in the membrane would be marked with red fluorescence. However, the newly synthesized PIN2 would display green color in the default PIN2-EosFP state (green; Jásik et al., 2013, 2016; Wiedenmann et al., 2004). By using PIN2-EosFP, we found that the degradation rate of PIN2 was not affected either exogenously (following ABA application) or endogenously (in the *cyp707a1,3* mutant) during 3 h after photoconversion, the RFP intensities of PIN2-EosFP in the root tip of either ABA-treated WT or *cyp707a1,3* seedlings remain similar to the control: remain approximately 40% of their original intensity (Figures 9A,C). The PIN2 degradation in *cyp707a1,3* seems even slightly slower than in the WT, as it possesses a higher ratio of RFP (but not significantly different). Similar result was obtained also from another transgenic line (Supplementary Figure 7). This result demonstrates that the PIN2 degradation was not accelerated by ABA.

Many membrane proteins, including PIN2, can be targeted to the lytic vacuole (LV) for degradation (Kleine-Vehn et al., 2008; Leitner et al., 2012). The acidification of the LV is prerequisite for the protein degradation. The Concanamycin A (ConcA), an inhibitor of the vacuole H^+^-ATPases, can reduce the acidification in LV and, thereby, inhibits the proteins degradation inside this organelle (Páli et al., 2004; Willige et al., 2011). Using 1 μM ConcA, we found that the PIN2-GFP aggregation in LV was slightly inhibited upon exogenous ABA treatment (Supplementary Figures 8A,B). However, a reduced PIN2-GFP signal was detected in the LV of *cyp707a1,3* (Supplementary Figure 8C). These results imply that the PIN2 degradation can be inhibited by the ABA. Moreover, ABA induces a decrease in PIN2 LV targeting, which was not completely consistent with the results obtained by the PIN2-EosFP photoconversion, suggesting that the PIN2 degradation was probably not affected by the ABA (Figures 9A,C).

In addition, the amount of the newly synthesized PIN2 was significantly reduced by ABA, as the GFP state of PIN2-EosFP following ABA treatment or in *cyp707a1,3* increased more slowly than in WT (Figures 9A,B). The relative *PIN2* expression in the root tip was significantly reduced following 8 μM ABA treatment or in *cyp707a1,3*, as indicated by qRT-RCR (Figure 6D). Hence, the decrease in the amount of newly synthesized PIN2 was at least partially caused by the suppression of *PIN2* expression. Thus, it is not fully clear whether the protein translation rate of PIN2 was affected. Therefore, it seems that ABA reduces the membrane PIN2 abundance mainly by reducing (slowing down) its *de novo* synthesis. Overall, the ABA reduces the PIN2 abundance by inhibiting its gene expression rather than accelerating of its degradation.

## Discussion

Considering that ABA is regarded as a stress plant hormone (Cutler et al., 2010; Zhang, 2014), we suppose that ABA may act as an intermediate between stressful conditions and root growth trajectory. Interestingly, previous researchers have shown that several adversities (including high salinity, hydrogen peroxide, soil hypoxia and bacteria) can lead to wavy root growth trajectory (Sun et al., 2008; Eysholdt-Derzso and Sauter, 2017; Zhou et al., 2018; Jimenez-Vazquez et al., 2020). This evidence clearly supports our assumption. Previously, Nie et al. (2014) investigated the characteristics of shrub roots in different soil conditions. The shrub root growth shows only a slight curvature in the soft soil but strong curvature in the hard soil (with rocks) (Nie et al., 2014). Soils with lower water content are typically harder than those with plenty of moisture. Hence, the wavy root growth trajectory in harder soil may increase the possibility of encountering moist areas with nutrients. Moreover, it has been recently reported that auxin could be involved in obstacle avoidance (Zhang and Friml, 2020). Thus, our data revealing a pivotal role for auxin in curved root growth might represent an alternative explanation for the curved root phenotype induced by hard soil-roots would apparently encounter obstacles more frequently in hard soil.

Previously, Sun et al. (2008) had reported the same phenotype induced by a high level of NaCl. Authors ascribed the curved phenotype to a compromised gravitropism. The tropic growth to other stimuli would become visible with the overwhelming decrease in the gravitropism (Sun et al., 2008). Interestingly, the parameter VGI that we used to quantify root curvature was suggested as a sensitive morphometric parameter to assess the defect in gravitropism (Grabov et al., 2005; Rigas et al., 2013; Wang et al., 2013). However, our results indicate that the agravitropism-like phenotype did not result from the compromised gravitropism. In our context, the gravitropic bending rate was not affected by 2 μM ABA (Supplementary Figure 9). However, this ABA concentration was sufficient to elicit the agravitropism-like phenotype (Figure 1). Consistently, several reports documented that ABA is unlikely to be a regulator of the root gravitropism (Lee et al., 1990; Moore, 1990; Young and Evans, 1996). Hence, we suggest that VGI reveals a different physiological aspect of the gravitropism reflected by gravitropic bending. VGI seems to be a long-term reaction or modulation of gravitropism that may last for several days. However, the gravitropic bending is usually completed within a maximum of 24 h (Hu et al., 2005; Perera et al., 2006).

**FIGURE 10:**
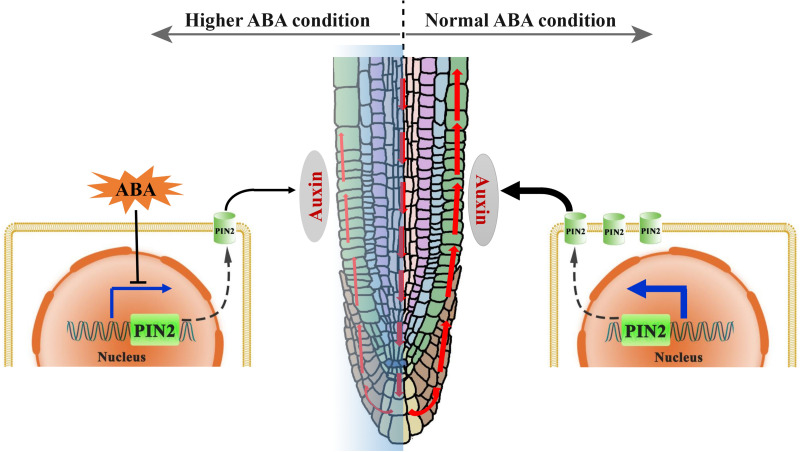
A proposed mechanistic model for the regulation of root growth trajectory in response to ABA. In normal (ambient) environmental condition, the auxin can be transported from the lateral root caps toward the elongation zone (EZ), mainly through a polar distributed auxin efflux carrier PIN2 (green cylinder in the membrane). A certain level of auxin in the EZ is required for the maintaining of a symmetric cell elongation. However, unfavorable environmental conditions could induce an elevated ABA level in root. Hence, higher ABA level suppresses the PIN2 transcription resulting thus in a lower PIN2 abundance, and leads consequently to the impairment of SAT (red upward arrowheads). An insufficiency in the auxin content in the EZ causes ultimately to an asymmetric cell elongation in this region, conferring thereby a wavy growth trajectory.

The crosstalk between ABA and auxin in the context of root growth were under extensive investigations. For examples: Munguia-Rodriguez et al. (2020) proved that excess auxin content may confer oversensitivity to root elongation induced by ABA; Thole et al. (2014) had demonstrated that root elongation in both *aux1* and *pin2* is insensitive to ABA. These together demonstrated that ABA could affect root growth through modulating auxin transport, and is in line with our main conclusions that ABA regulates root growth trajectory via auxin transporter (PIN2; Figure 10). The above cited researches shade lights on the correlations between hormonal crosstalk (ABA and auxin) and root elongation. Our work is dedicated to studying the role of hormonal crosstalk in root growth trajectory. We provide evidence that ABA regulates the root growth trajectory/direction by modulating PIN2 abundance (Figure 5). Considering the versatile function of ABA in the stress response, our results indicate that PIN2 may serve as an intermedia between the stress conditions and root growth trajectory. Consistently, the role of PIN2 in growth adaptability has been emphasized (Remy et al., 2013). Moreover, we demonstrate the importance of epidermal PIN2 in modulating the root growth trajectory (Figure 6), which had never been previously documented.

We notice that the activity of the GL2 promoter was much weaker than the PIN2 native promoter because even in the strongest line selected among 20 independent GL2:PIN2-EosFP/*pin2-1* T_3_ lines according to their *PIN2* expression, the PIN2-EosFP intensity was much weaker than the average recorded in the epidermis of PIN2:PIN2-EosFP/*pin2-1*. However, even driven by a frail promotor, GL2:PIN2-EosFP shows a substantial increase in VGI from 0.49 to 0.77, representing an approximately 60% difference between *pin2-1* and WT (Figure 8). Thus, the epidermal PIN2 bears (assumes) a major responsibility in controlling the root trajectory/direction. This is a plausible revelation because the epidermis is located in the root outermost layer. Accordingly, such physiological processes, including asymmetric cell elongation during tropic bending, would certainly be exacerbated in the epidermis.

We noticed that after transfer, the lateral roots growth was apparently promoted by ABA, since both of the lateral roots number and length were increased in the 1/2 MS medium containing ABA (Figure 1 and Supplementary Figure 10). Recently, Jimenez-Vazquez et al. (2020) have reported that a beneficial rhizobacterium *Achromobacter* sp. 5B1 could elicit a similar phenotype as ABA does. Interestingly, the bacteria also promotes lateral root formation which may due to increased wavy frequency along growth trajectory (Jimenez-Vazquez et al., 2020). Although ABA promotes lateral roots elongation and formation as well as the wavy amplitude of growth trajectory (Figure 1). Meanwhile, promotion on lateral roots does not occur in the curved part of the root (a root part grown after transfer). Therefore, the mechanism underlines the ABA promoting effect on the lateral root formation might be different from that reported by Jimenez-Vazquez et al. (2020).

In conclusion, we revealed in this report that the exogenous and endogenous ABA accumulation could result in the root agravitropism-like growth pattern. The agravitropism-like phenotype resulted from the reduced auxin content of SAT caused by ABA (Figure 10). ABA mitigation of the SAT by decreasing the PIN2 abundance, and the latter (reduction in PIN2 abundance) was attributable to the suppression of its gene expression rather than the acceleration of its degradation (Figure 10). Altogether, ABA suppresses the PIN2 expression, inhibiting thereby the SAT (Figure 10). This attenuation of SAT would ultimately elicit the agravitropism-like phenotype.

## Data Availability Statement

The original contributions presented in the study are included in the article/Supplementary Material, further inquiries can be directed to the corresponding author/s.

## Author Contributions

QX and WC: conceptualization and writing. QX, WC, and JE: methodology and design. QX, JE, XP, and HC: experiments. QX, XP, and HC: investigation. WC: funding acquisition and supervision. QX and JE: resources. All authors contributed to the article and approved the submitted version.

## Conflict of Interest

The authors declare that the research was conducted in the absence of any commercial or financial relationships that could be construed as a potential conflict of interest.

## Footnotes

1 https://imagej.nih.gov/ij/

2 https://www.olympus-lifescience.com.cn/

## Abbreviations

ABA: abscisic acid
ABF: ABRE BINDING FACTOR
BSA: Bovine Serum Albumin
ConcA: Concanamycin A
EZ: elongation zone
HPLC: high-performance liquid chromatography
MS: Murashige and Skoog
PBS: Phosphate buffer saline
PIN: PIN-FORMED
PI: propidium iodide
QC: quiescent centre
SAT: shootward auxin transport
VGI: vertical growth index.
