# Supplementary material for: Abscisic Acid Regulates the Root Growth Trajectory by Reducing Auxin Transporter PIN2 Protein Levels in Arabidopsis thaliana
Source: Front Plant Sci. 2021 Mar 4;12:632676. doi: 10.3389/fpls.2021.632676 (PMC7982918; doi:10.3389/fpls.2021.632676)
Supplement: Supplementary file 1 [file Data_Sheet_1.docx]

**Supplementary Figures**


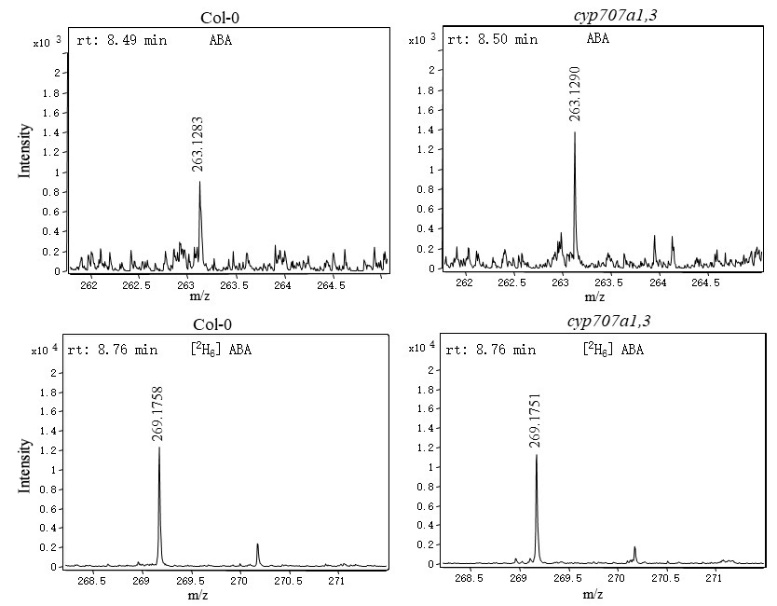


**Supplementary Figure 1:** A typical mass spectrometry (MS) of ABA and its interrnal stander [^2^H_6_] ABA from Col-0 and *cyp707a1,3* root extracts. Numbers adjacent to each peak indicate m/z of ABA (panels in left colum) or [^2^H_6_] ABA (panels in right colum), The letters “rt” stand for retention time, respectively.


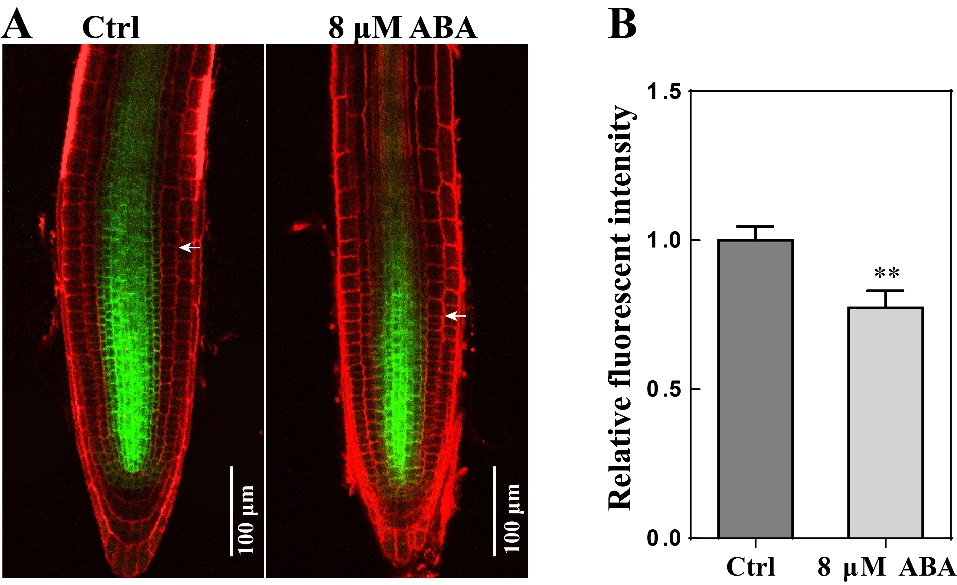


**Supplementary Figure 2:** Representative confocal images of PIN1::PIN1-GFP in control and upon ABA treatment.

**A**: Seedlings of PIN1::PIN1-GFP following ABA treatments. 5-d old seedlings grown on 1/2 MS were transferred onto repective 1/2 medium with or without 8 μM ABA. Roots were observed with confocal microscopy 1 d after transfer, using 488 nm wave length to excit GFP (green signal) and 559 nm to excit PI (red signal). The white arrowhead indicates the bundary of meristem zone (MZ). PI stands for propidium iodide

**B**: Raletive fluorescent intensities of PIN1-GFP following same treatments as in panel A. Fluorescent intensities were normalized to the control. Each bar data of the histogram represents the mean of relative fluorescent intensities from 30 independent measurements ±SE.


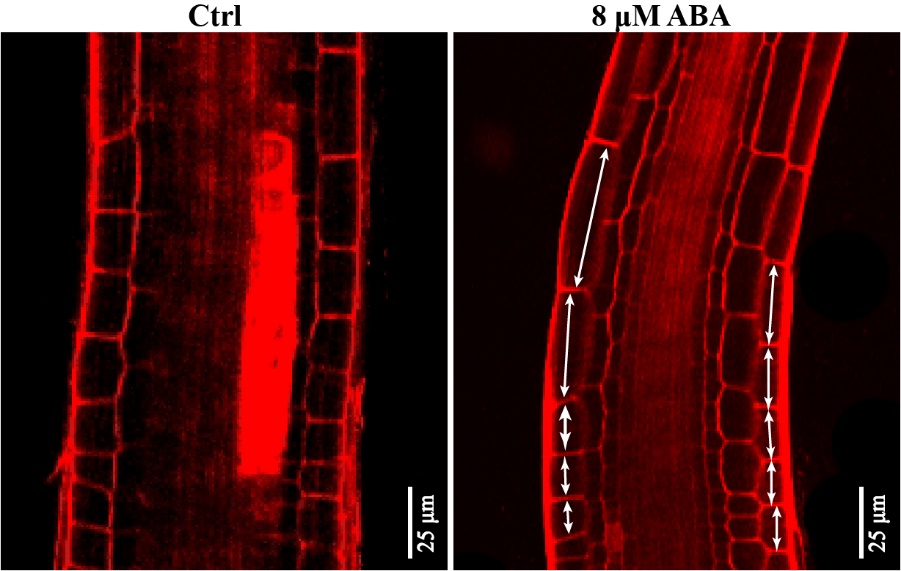


**Supplementary Figure 3:** Typical growth rate of epidermal cells in the bending site of control and following ABA treatment. 5-dold Col-0 seedlings were treated with ABA for an additional day. Roots were stained with PI before microscopy. 559 nm laser was used to excit PI dye. The double-headed arrows indicate the cell length in concave and convex shape. PI means propidium iodide.


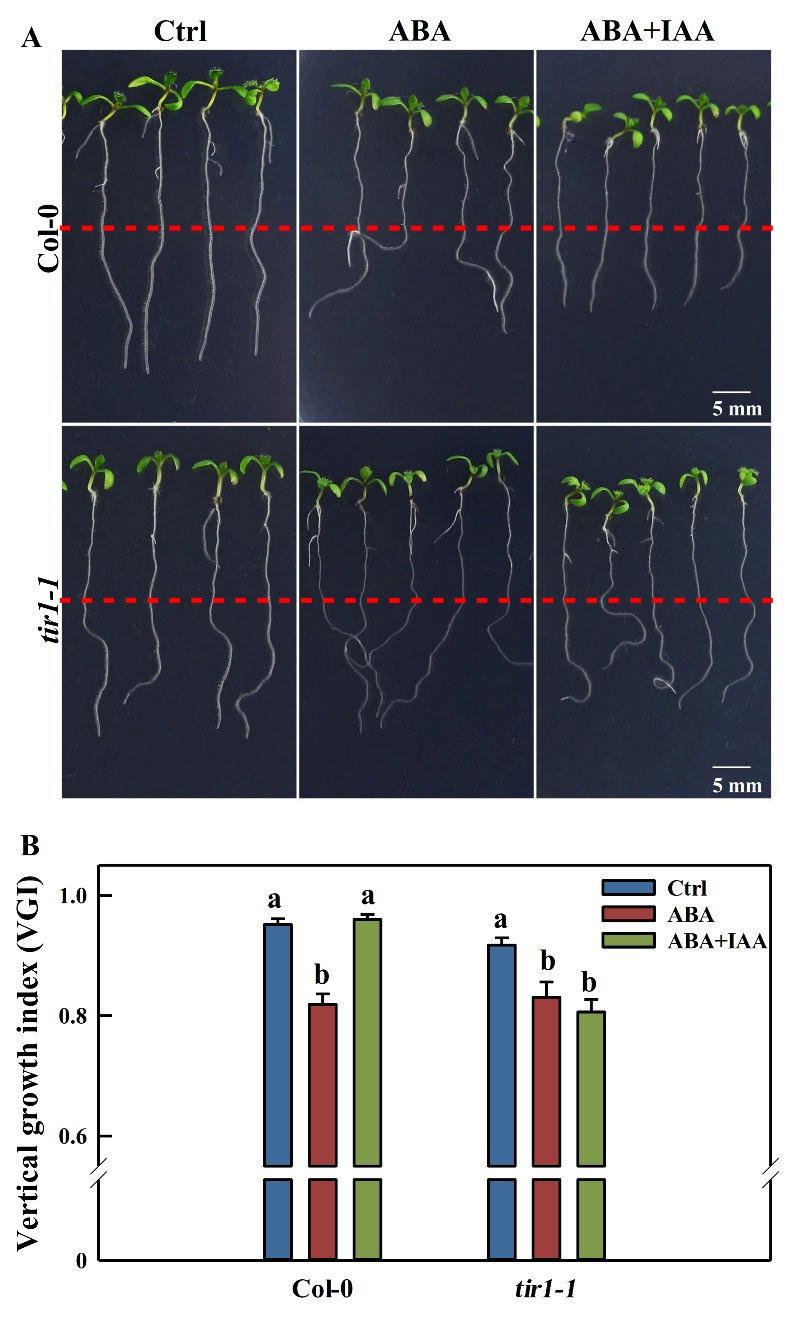


**Supplementary Figure 4:** Root vertical growth index (VGI) of Col-0 and *tir1-1* following ABA treatment alone or together with IAA.

**A**: Seedlings of Col-0 and *tir1-1* following phytohormonal treatments. 5-d old seedlings grown on 1/2 MS were transferred onto repective 1/2 medium without hormonal addition (Ctrl), with 8 μM ABA or 8 μM ABA together with 5 nM IAA. After 3 d of treatments, seedlings were pohotographed.

**B**: Root VGI of Col-0 and *tir1-1* following same treatments as in panel **A**. The VGI was measured accroding to the method described in Figure 1B. Each bar data of the histogram represents the mean value of root VGI obtained from 30 independent seedlings ±SE.


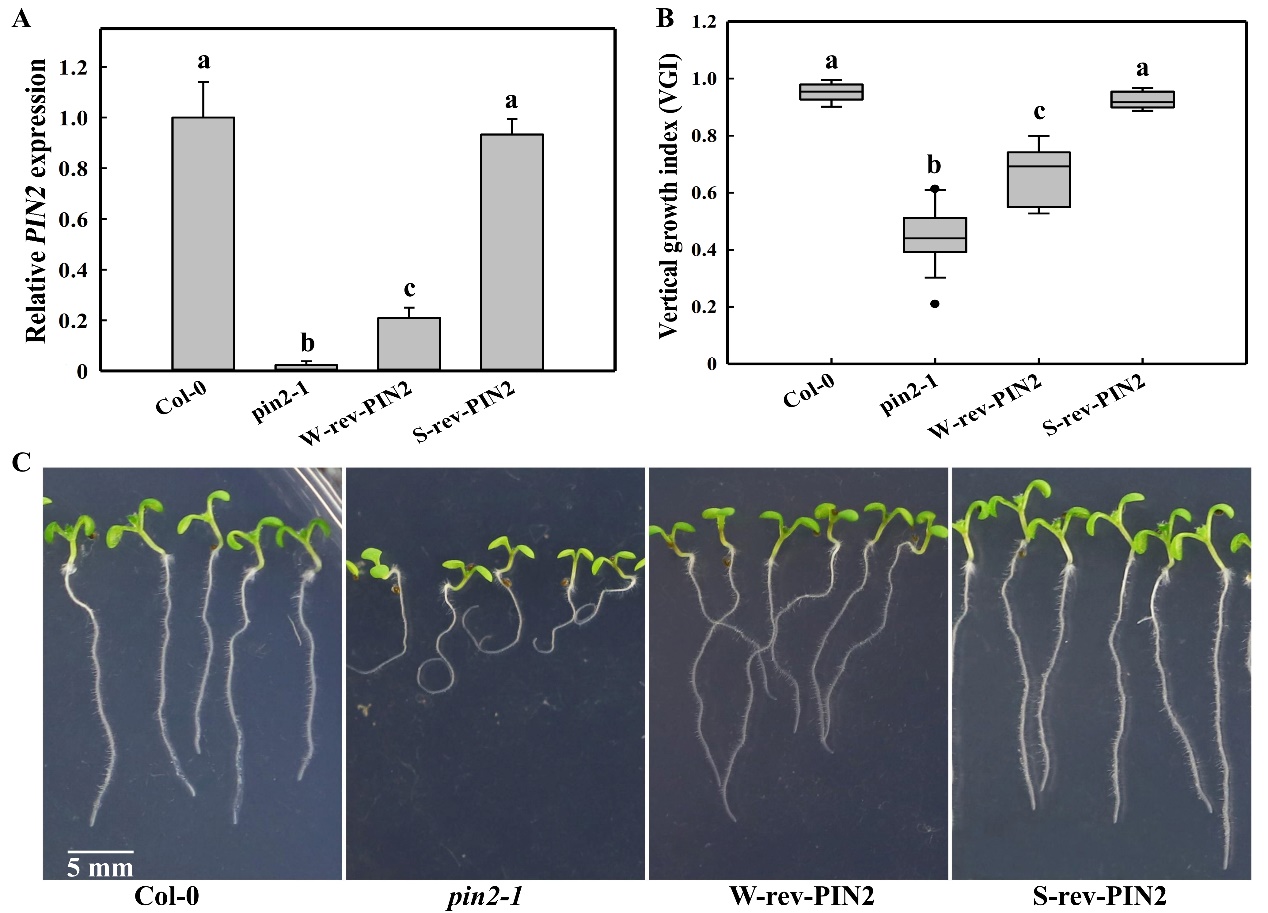


**Supplementary Figure 5:** Root VGI of Col-0, *pin2-1* and two *PIN2* recovery lines W-rev-PIN2 and S-rev-PIN2.

**A**: Relative *PIN2* expression levels in root tips of Col-0, *pin2-1*, W-rev-PIN2 and S-rev-PIN2. PIN2 expression levels were evaluated in 5-d old seedlings. The W-rev-PIN2 and S-rev-PIN2 are two independent T_3_ PIN2 recovery lines, obtained (or generated) by transforming PIN2::PIN2-EosFP construct into *pin2-1* background. Two lines were selected according to their relative expression levels, where W-rev-PIN2 posseses a relatively lower *PIN2* expression compare to WT (Col-0) and S-rev-PIN2 represents a line with a similar *PIN2* expression level to WT.

**B**: Box plot showing the VGI of Col-0, *pin2-1*, W-rev-PIN2 and S-rev-PIN2. Seedlings were gown on 1/2 MS medium, and were photographed 5 d later. Root VGI were measured as described in Figure 1A. n > 30.

**C**: Representative images of Col-0, *pin2-1*, W-rev-PIN2 and S-rev-PIN2 .


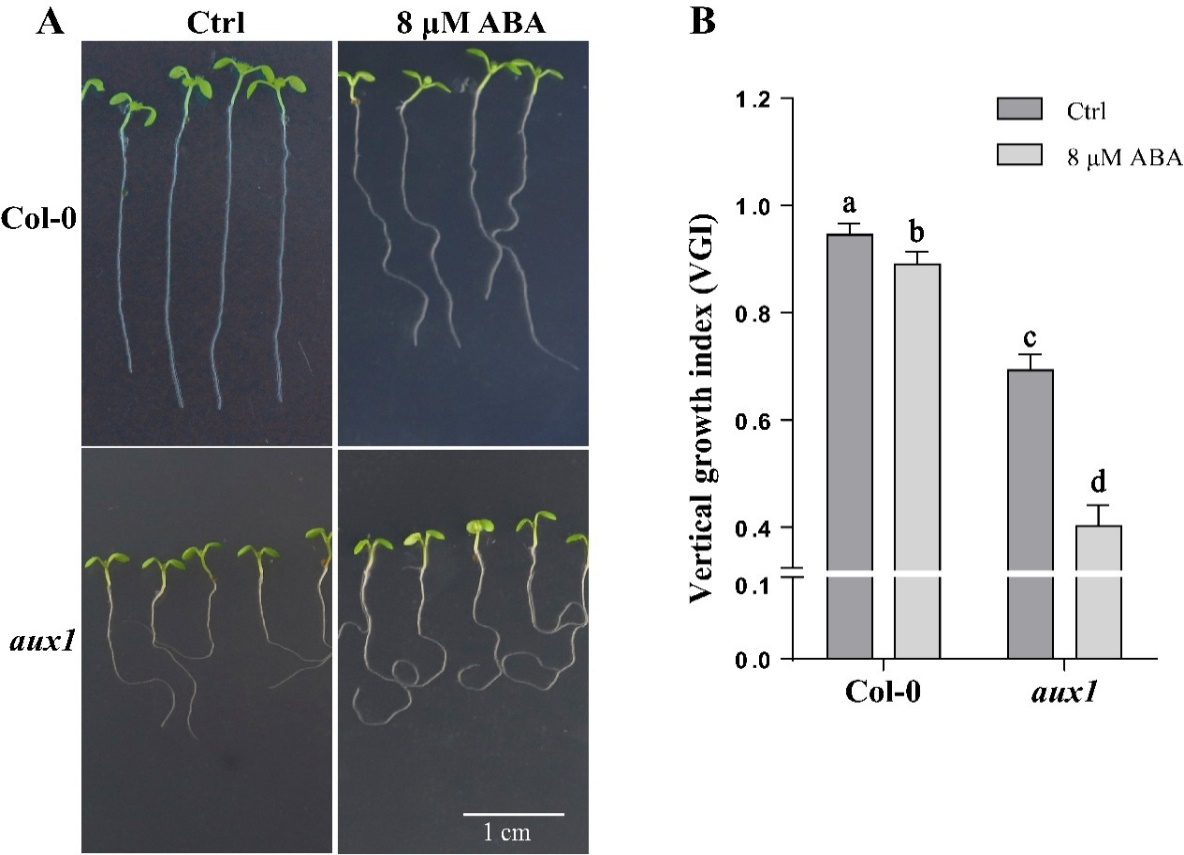


**Supplementary Figure 6:** Root VGI of Col-0 and *aux1* for control and following ABA application.

**A**: Representative images of Col-0 and *aux1* seedlings of control and following ABA application. 5-d-old seedlings were either untreated (ctrl) or treated without 8 μM ABA for 3 d. The root were photographed 3 days after treatment.

**B**: VGI of Col-0 and *aux1* following same treatments as in panel A. Each bar data represents the mean value of the root VGI measured on 30 different seedlings ±SE.


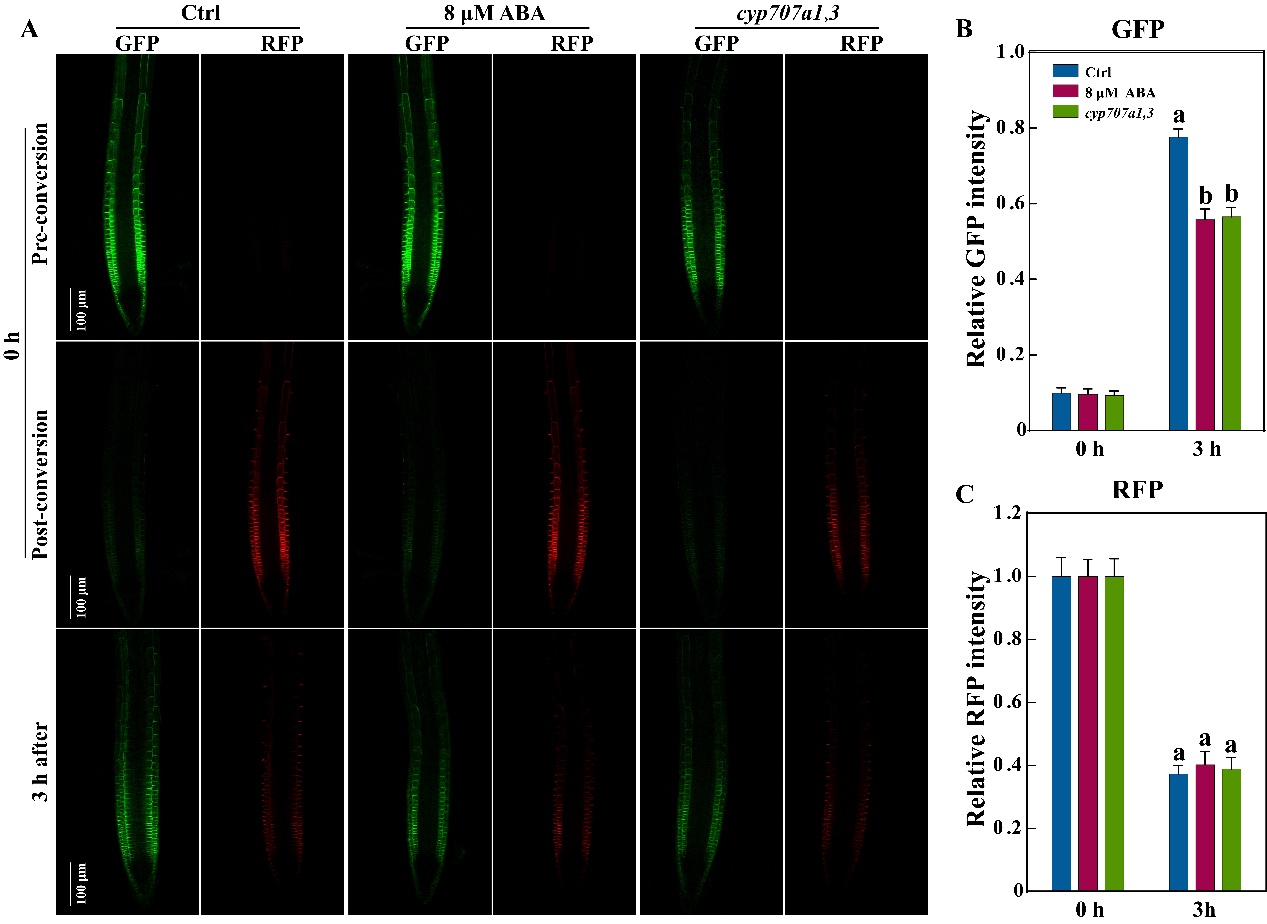


**Supplementary Figure 7:** PIN2 synthesis and degradation in Col-0, ABA-treated Col-0 and the *cyp707a1,3* double mutant as revealed by photoconversion of another PIN2-EosFP line. This PIN2-EosFP is another independent transgenic line possesses strong PIN2-EosFP expression. For detailed explanation about this figure please refer to the figure 9 legend.


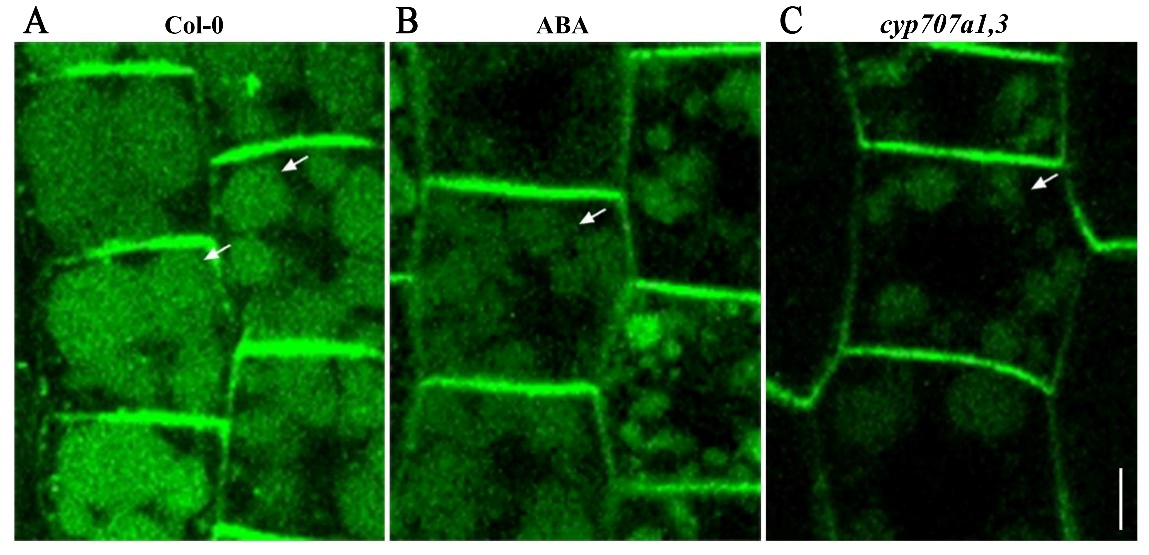


**Supplementary Figure 8:** Concanamycin A (ConcA) induced PIN2-GFP accumulation in lytic vacuole (LV) in root epidermis of untreated Col-0 (Ctrl), ABA-treated Col-0 and *cyp707a1,3*.

**A**: PIN2-GFP in Col-0 background. 5-day-old seedlings grown on 1/2 MS medium were transferred into 1/2 liquid MS medium containing 1 μM ConcA. After 3 h of treatment, seedlings were ready for confocal microscopy observation.

**B**: PIN2-GFP (Col-0) following ABA treatment. The same seedlings discribed in panel A were transferred into 1/2 liquid MS medium contaning 1 μM ConcA and 8 μM ABA.

**C**: PIN2-GFP in *cyp707a1,3* background. Following the same treatment described in panel A. the scaling bar equals to 5 μm.


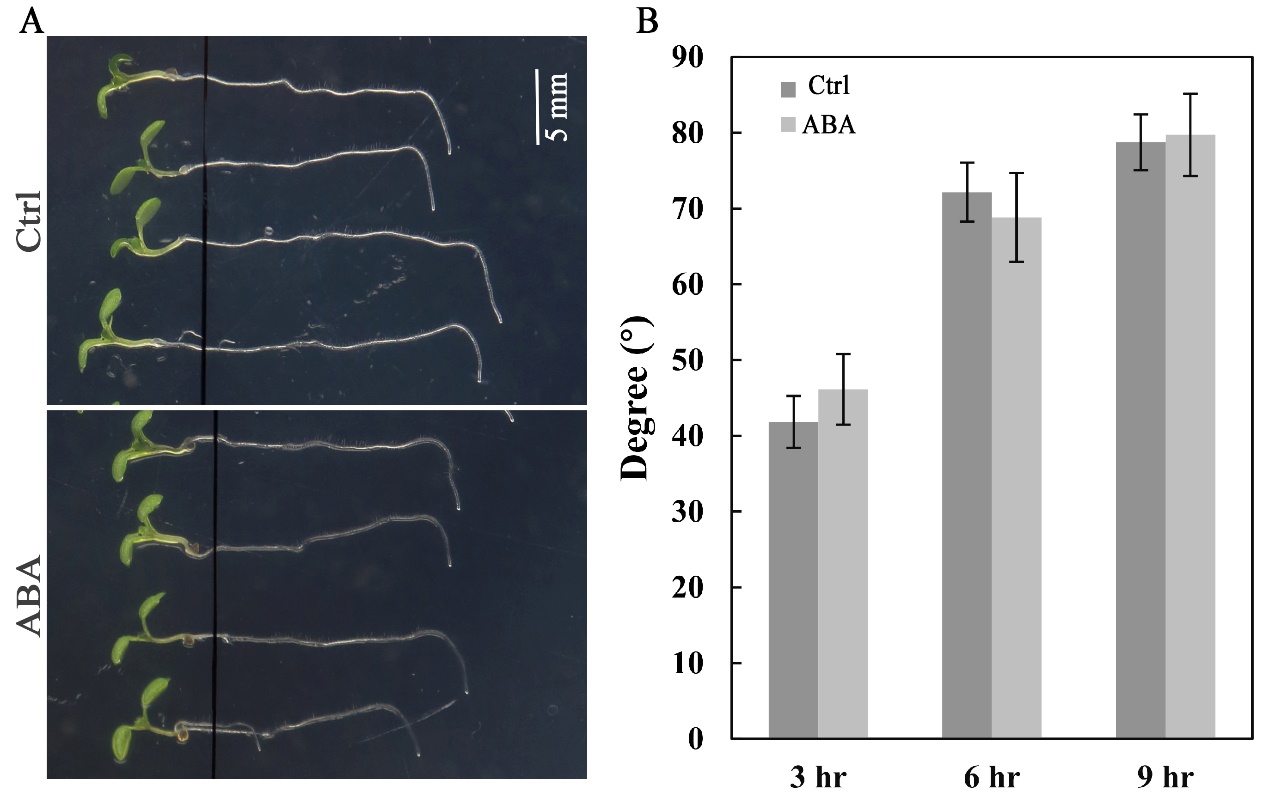


**Supplementary Figure 9:** Root gravitropic bending rates of control following ABA treatment.

**A**: Representative photos of roots from control and ABA-treated seedling after gravistimulation. 5-d-old Col-0 seedlings were transffered on 1/2 MS medium in the absence (without ABA) or the presence of ABA (2 μM). A single day after tranplantation, roots were horizontally placed and photos taken at 3, 6 and 9 h time points. The photos display seedling 6 h after gravistimulation.

**B**: Root gravitropic beinding rates of control or following 2 μM ABA treatment. Gravitropic bending rates were measured according to the photos described in panel A. Each bar data represents the mean value of gravitropic bending rate from 30 different seedlings ±SE


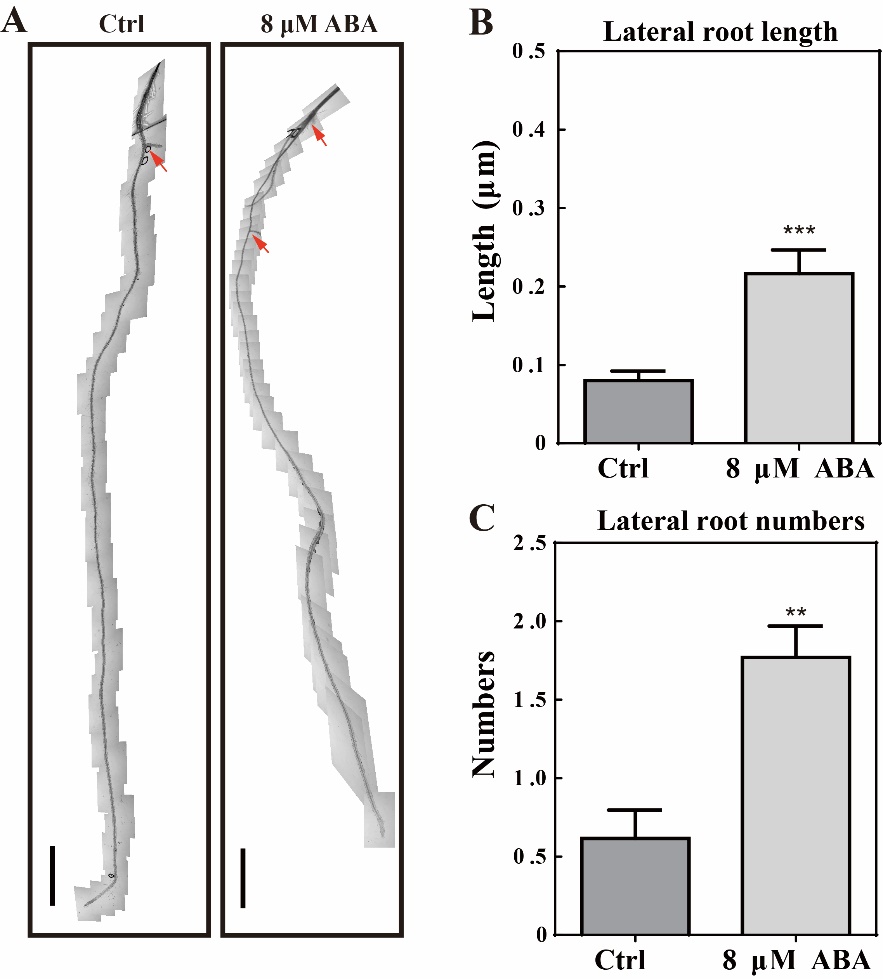


**Supplementary Figure 10:** Lateral roots (or primordium) of control and ABA-treated seedlings

**A**: Merged microscope images of roots obtained from control and ABA-treated seedlings. 5-d-old Col-0 seedlings were untreated (Ctrl) or tretaed with 8 μM ABA. The microscope images were taken frame by frame along the root axis and were merged into a picture to show the root primordium (leteral roots). The red arrow head indicates the leteral root or root primordium. Bar = 500 μm.

**B**: Lateral root (or primordium) of control and following ABA treatment. Each bar data represents the mean values from 30 independent seedlings ±SE

**C**: Average lateral root length of control and following ABA treatment. For panels B and C, each bar data represents the mean length of lateral roots measured on 30 independent seedlings ±SE.
